# Defects in the necroptosis machinery are a cancer resistance mechanism to checkpoint inhibitor immunotherapy

**DOI:** 10.1136/jitc-2024-010433

**Published:** 2025-05-08

**Authors:** Anna Sax, Peter May, Stefan Enssle, Nardine Soliman, Tatiana Nedelko, Giada Mandracci, Fabian Stögbauer, Laura Joachim, Christof Winter, Florian Bassermann, Katja Steiger, Nadia El Khawanky, Hendrik Poeck, Simon Heidegger

**Affiliations:** 1 Department of Medicine III, Technical University of Munich, TUM School of Medicine and Health, Munich, Germany; 2 Center for Translational Cancer Research (TranslaTUM), Technical University of Munich, TUM School of Medicine and Health, Munich, Germany; 3 Institute of Pathology, Technical University of Munich, TUM School of Medicine and Health, Munich, Germany; 4 Institute of Clinical Chemistry and Pathobiochemistry, Technical University of Munich, TUM School of Medicine and Health, Munich, Germany; 5 German Cancer Consortium (DKTK), Partner-site Munich and German Cancer Research Center (DKFZ), Heidelberg, Germany; 6 Bavarian Cancer Research Center (BZKF), Munich & Regensburg, Germany; 7 Department of Internal Medicine III, University Hospital Regensburg, Regensburg, Germany; 8 Leibniz Institute for Immunotherapy (LIT), Regensburg, Germany; 9 Center for immunomedicine in transplantation and oncology (CITO), Regensburg, Germany

**Keywords:** Immunotherapy, Tumor microenvironment - TME, Immune Checkpoint Inhibitor, T cell

## Abstract

**Background:**

Immune checkpoint inhibitors (ICIs) of programmed cell death protein-1 (PD-1) or cytotoxic T-lymphocytes-associated protein 4 (CTLA-4) reinvigorate strong polyclonal T-cell immune responses against tumor cells. For many patients, these therapies fail because the development of spontaneous immune responses is often compromised, as the tumor microenvironment (TME) lacks proinflammatory signals resulting in suboptimal activation of antigen-presenting cells (APCs). Necroptosis is a special form of programmed cell death associated with leakage of inflammatory factors that can lead to APC maturation. However, it is unclear to which extent functional necroptosis in tumor cells contributes to ICI immunotherapy.

**Methods:**

With genetically engineered tumor cell lines that lack specific components of the necroptosis machinery (mixed lineage kinase domain-like pseudokinase (MLKL), receptor interacting protein kinase 3 (RIPK3)), we addressed the importance of necroptotic tumor cell death for the efficacy of ICI immunotherapy in murine models. Preclinical data were aligned with genome-wide transcriptional programs in patient tumor samples at diagnosis and during ICI treatment for the activity of these pathways and association with treatment outcome.

**Results:**

Mice bearing MLKL-deficient or RIPK3-deficient tumors failed to control tumor growth in response to anti-PD-1/anti-CTLA-4 immunotherapy. Mechanistically, defects in the necroptosis pathway resulted in reduced tumor antigen cross-presentation by type 1 conventional dendritic cells (DCs) in tumor-draining lymph nodes, and subsequently impaired immunotherapy-induced expansion of circulating tumor antigen-specific CD8^+^ T cells and their accumulation and activation in the TME. In vitro, co-culture of tumor cells undergoing necroptotic but not apoptotic programmed cell death resulted in increased uptake by phagocytic cells, associated with maturation and activation of DCs. Treatment of tumors with the epigenetic modulator azacytidine enhanced intrinsic transcriptional activity of the necroptosis machinery, and hence their susceptibility to ICI immunotherapy. In humans, transcriptome analysis of melanoma samples revealed a strong association between high expression of *MLKL* and prolonged overall survival and durable clinical response to immunotherapy with anti-PD-1 and/or anti-CTLA-4 checkpoint inhibitors.

**Conclusions:**

Defective necroptosis signaling in tumor cells is a cancer resistance mechanism to ICI immunotherapy. Reversion of epigenetic silencing of the necroptosis pathway can render tumors susceptible to checkpoint inhibition.

**WHAT IS ALREADY KNOWN ON THIS TOPIC**

- The lack of proinflammatory signals in the tumor microenvironment to reach a threshold for sufficient immune cell activation is one of the reasons for low response rates to immune checkpoint inhibitors in many patients with cancer.

**WHAT THIS STUDY ADDS**

- Necroptosis, a form of inflammatory programmed cell death, in tumor cells can drive cross-presentation of tumor antigens by specialized dendritic cells and priming of antitumor T cell immunity. Defects in the necroptosis machinery are a mechanism how tumor cells harbor resistance to checkpoint inhibitor tumor immunotherapy.

**HOW THIS STUDY MIGHT AFFECT RESEARCH, PRACTICE OR POLICY**

- Our data warrant further exploration of how the activity of the necroptosis pathway in tumor cells may be used as a predictive biomarker for certain immunotherapies, and how therapeutic activation of the necroptosis machinery may overcome resistance to checkpoint inhibitor therapy in patients with immunologically “cold” tumors.

## Background

 Immunotherapeutic concepts including immune checkpoint inhibitors (ICIs) of cytotoxic T-lymphocytes-associated protein 4 (CTLA-4) and programmed cell death protein-1 (PD-1) have dramatically changed clinical practice in cancer treatment. These therapies reinvigorate T-cell responses against tumor cells. However, for many patients these therapies fail, as the initial development of spontaneous immune responses is often compromised by the immunosuppressive tumor milieu or insufficient levels of tumor antigen to reach the threshold for T-cell recognition. A prerequisite for the initiation of tumor-specific adaptive T-cell immune responses is that specialized antigen-presenting cells (APCs)—particularly dendritic cells (DCs)—take up, process and present tumor-associated antigens to cytotoxic T cells. Such cross-priming of tumor-specific T cells has been shown to be dependent on DC maturation mediated by type I interferon (IFN-I).^1^ In contrast to microbial infections, the tumor microenvironment (TME) often lacks proinflammatory signals, resulting in suboptimal DC activation.

Under certain circumstances, tumor cells can undergo special forms of programmed tumor cell death that favor recognition and elimination by the immune system.^2^ Such immunogenic cell death (ICD) has been shown in response to treatment with certain chemotherapeutic agents (oxaliplatin, doxorubicin) or radiation. One of the characteristics of ICD seems to be the spatiotemporally defined release of proinflammatory factors called danger-associated molecular patterns (DAMPs), which can lead to DC maturation via stimulation of innate pattern recognition receptors. There are various forms of programmed cell death, each responding to cellular insults but differing in their underlying molecular mechanisms.^2^ Apoptosis occurs after activation of a family of proteases termed caspases (finally executed via caspase-3), and the clearance of apoptotic debris is widely considered silent (non-immunogenic), associated with tolerogenic signaling. In contrast, necroptosis, mediated via its executioner protein MLKL (mixed lineage kinase domain-like pseudokinase), is considered immunogenic as it results in the leakage of DAMPs during programmed cell death. DAMPs comprise both constitutively present cellular components only released upon cell membrane disintegration as well as factors that are de novo synthesized induced by proinflammatory transcription factors in dying cells.^3^ DAMPs that have been associated with but are not unique to necroptotic cell death include exposure of calreticulin on the cell surface as well as release of adenosine triphosphate (ATP) and high mobility group box protein 1 (HMGB1).^2^

The known molecular mechanisms of necroptosis mostly derive from studies in the context of infectious pathogens. In such cases, necroptosis is often characterized as a “backup mechanism” for cell death when caspases (particularly caspase-8) are inhibited in virus-infected (or malignant) cells.^4^ Necroptosis can be initiated via engagement of (1) death receptors (including TNF receptor-1), (2) specific innate pathogen recognition receptors including nucleic acid sensors, and (3) IFN-I signaling.^3 5^ In the absence of inhibitory caspase-8, subsequently activated receptor interacting protein kinase (RIPK)-1 via its RHIM domain can form a complex with RIPK3, which phosphorylates and oligomerizes MLKL. Recently, other RHIM domain-containing proteins such as Z-DNA binding protein 1 (ZBP1) and TRIF, have been shown to be able to recruit RIPK3 for MLKL activation.^6 7^ MLKL multimers then translocate to the plasma membrane, where they interact with phospholipids and form pores, inducing inflammatory cell death with the release of a plethora of DAMPs.

While there is a broad consensus about the immunogenicity of necroptotic tumor cell death and that therapeutically engaged necroptosis signaling can enhance antitumor T-cell immunity,^3^ it largely remains unclear to which extent these ICD mechanisms play a role in spontaneous tumor immunosurveillance or clinically practiced immunotherapies. We here define insufficient tumor cell-intrinsic activity of the necroptosis machinery as a cancer resistance mechanism to clinically established ICIs of CTLA-4 and PD-1.

## Methods

### Mice

Female C57BL/6J mice were purchased from Janvier, BALB/c mice from Charles River. Mice were between 6 and 8 weeks old at the onset of experiments and were maintained in specific pathogen-free conditions on a 12 hours light–dark cycle and a constant temperature of 24°C. Animal studies were approved by the local regulatory agency (Regierung von Oberbayern, Munich, Germany; application ID ROB-55.2–2532.Vet_02-19-159).

### Media and reagents

Roswell Park Memorial Institute (RPMI)-1640 medium (Invitrogen) and Dulbecco’s Modified Eagle’s Medium (DMEM) (Invitrogen) were supplemented with 10% (v/v) fetal calf serum (FCS) (Gibco), 3 mM L-glutamine, 100 U/mL of penicillin and 100 µg/mL of streptomycin (all from Sigma-Aldrich). Iscove's Modified Dulbecco's Medium (IMDM) and F12 (both Cytica) were mixed at a 2:1 ratio and supplemented with 10% (v/v) FCS, 100 U/mL penicillin 100 µg/mL streptomycin, 5 ng/mL epidermal growth factor (EGF) (PeproTech), 400 ng/mL hydrocortisone and 5 mg/mL insulin (both Sigma-Aldrich). To induce necroptosis, cells were treated for 24 hours with 20 ng/mL TNFalpha (PeproTech), 1 µM LCL 161 (Sigma) and 20 µM zVAD FMK (Invivogen), or 30 µM Necrostatin-1 (Biovision) to inhibit necroptosis. Cell death was assessed by staining with fluorescein isothiocyanate (FITC)-labeled annexin V and propidium iodide (both BioLegend). Azacytidine (AZA, Celgene) and 5-aza-2′-deoxycytidine (5-AD, Janssen) were obtained via the hospital pharmacy of Klinikum rechts der Isar at Technical University Munich, diluted in dimethyl sulfoxide (DMSO) for working stocks and were used in the indicated concentrations.

### Cell lines and CRISPR-Cas9-mediated genome editing

The B16 murine melanoma cell line expressing full-length chicken ovalbumin (here referred to as B16.OVA) was cultured in complete DMEM. CT26 and Panc02.OVA cells were cultured in complete DMEM or RPMI medium, respectively. MOC1 and MOC2 cells were maintained in IMDM/F12 medium. All cells were cultured at 37°C with 5% CO_2_. Polyclonal gene (*Mlkl, Ripk3*)-deficient B16.OVA, CT26 and Panc02.OVA cells were engineered using the CRISPR-Cas9 system. To achieve high efficiency of gene deletion, two guide RNAs (gRNA) were used. After annealing of Alt-R CRISPR-Cas9 crRNA and tracrRNA (both Integrated DNA Technologies) to gRNAs, CRISPR/Cas9 ribonucleoprotein complexes consisting of gRNAs and TrueCut Cas9 Protein v2 (Invitrogen) were electroporated into cells using the 4D-Nucleofector X Unit (Lonza). Gene deficiency was identified by immunoblotting, functional assays, and sequencing. Target sequences of all used gRNAs can be found in the Online supplemental material and methods section.

### Murine bone marrow-derived dendritic cell and macrophage co-cultures

Murine bone marrow-derived dendritic cells (BMDCs) and macrophages (BMDMs) were generated by culturing bone marrow cells in very low endotoxin RPMI medium supplemented with 10% (v/v) FCS, 100 U/mL penicillin, 100 µg/mL streptomycin, 0.1% β-mercaptoethanol (50 mM) (Thermo Fisher) and granulocyte-macrophage colony-stimulating factor (M-CSF) (20 ng/mL) for BMDCs or M-CSF (40 ng/mL) for BMDMs (both PeproTech). For BMDC co-cultures, 10^4^ MOC1 cells were seeded in a 96-well plate and treated with TS, TSZ or TSZ+Nec-1 for 24 hours. Cells were co-cultured with 2×10^4^ BMDCs for 24 hours. For BMDMs, 5×10^5^ MOC1 cells were seeded in a 6-well plate and were fluorescently labeled with carboxyfluorescein succinimidyl ester (CFSE) (2,5 µM, Invitrogen) for 20 min in phosphate-buffered saline (PBS). After washing, cells were treated with TSZ or TSZ+Nec-1 for 24 hours and then co-cultured with 3.5×10^5^ BMDMs for 24 hours. Activation of BMDCs and uptake of CFSE-labeled tumor cell debris by BMDMs were determined by flow cytometry.

### Flow cytometry

Cell suspensions were stained in PBS with 1% FCS. Fluorochrome-coupled antibodies were purchased from eBioscience or BioLegend. In all experiments, a Live/Dead fixable cell stain (Invitrogen) was used to exclude dead cells, and an anti-CD16/CD32 antibody (BioLegend) was applied to block non-specific binding via Fc receptors for 20 min at 4°C. For intracellular cytokine staining, the Foxp3 Transcription Factor Fixation/Permeabilization Kit (eBioscience) was used. Tumor model antigen OVA-specific T cells were defined as live CD3^+^, CD4^−^ and SIINFEKL-H-2K^b+^ using iTAg major histocompatibility complex (MHC)-I murine tetramers detecting SIINFEKL-specific CD8^+^ T cells (MBL). Conventional type 1 DC (cDC1) were defined as CD11c^+^, CD11b^−^, CD8^+^, CD103^+^, MHCII^+^. Data were acquired on a FACSCanto II (BD Biosciences) and analyzed using FlowJo software (Tree Star).

### Tumor challenge and treatment

For tumor challenge with therapeutic treatment, mice were injected subcutaneously with 1.5×10^6^ Panc02.OVA cells, 10^6^ CT26 cells, 2×10^6^ MOC1 cells or 2.4×10^5^ B16.OVA cells in the right flank on day 0. Intraperitoneal treatment with anti-CTLA-4 (clone 9H10), anti-PD-1 (clone RMP1-14) or isotype control (all BioXCell) was performed on days 6 (200 µg), 9 (100 µg) and 12 (100 µg) in the B16.OVA tumor model or days 5 (200 µg), 8 (100 µg) and 11 (100 µg) in CT26 tumors. Panc02.OVA tumors were treated with anti-CTLA-4 or anti-PD-1 on days 3 (200 µg), 6 (100 µg) and 9 (100 µg). In the MOC1 tumor model, mice were injected with anti-CTLA-4 on days 14 (200 µg), 18 (100 µg), and 22 (100 µg). For analysis of OVA-specific T cells in the peripheral blood of mice inoculated with B16.OVA cells, blood was drawn from the facial vein and collected in EDTA microvette tubes (Sarstedt). After transfer to a 96-well plate and centrifugation (400×g, 5 min), cells were resuspended in red blood cell lysis buffer (Invitrogen), incubated for 5 min at room temperature, and PBS was added before another centrifugation step (400×g, 5 min). The lysis was repeated twice, and cells were then stained for flow cytometry. Mice were euthanized when the maximum tumor diameter exceeded 15 mm or other termination criteria were met according to standard legal procedure (responsible state office Regierung von Oberbayern).

### Preparation of cell suspensions from tumors and draining lymph nodes

At day 14 after B16.OVA tumor inoculation, some mice were sacrificed and tumors as well as tumor-draining (inguinal) lymph nodes were removed using forceps and surgical scissors. Tumors and lymph nodes were minced and homogenized by sequentially filtering through a 100 µm and a 70 µm nylon strainer (BD Bioscience). The cell suspensions were washed in PBS before subsequent analysis.

### Phospho-MLKL immunohistochemistry

Phospho-MLKL (pMLKL) immunohistochemistry (IHC) was performed by adapting a previously published methods protocol.^8^ In brief, murine tumor tissue was fixed in 10% neutral-buffered formalin solution (Sigma) for 72 hours, dehydrated under standard conditions using a tissue processor (Leica ASP300S) before being embedded in paraffin and cut to 2 μm-thin sections with a rotary microtome (HM355S, Thermo Fisher Scientific). IHC was conducted with a Bond RXm system (Leica, all reagents from Leica) with a primary antibody against pMLKL pS345 (dilution 1:5,000, Abcam: ab196436). Deparaffinization was performed (Leica Kit de-wax), followed by rehydration with alcohol washing and pretreatment with citrate buffer at pH 6 for 20 min to induce epitope retrieval. The samples were incubated with primary antibody for 15 min at room temperature and detected using the polymer refine detection kit. Counterstaining was performed with hematoxylin. IHC slides were imaged using a Leica Aperio imager AT2 at a 40× magnification, and whole slide images were analyzed with QuPath. Positive tumor cells were detected with the positive cell detection tool and the threshold was set to 0.25 for cytoplasmic 3,3′-diaminobenzidine (DAB) optical density (OD) mean intensity. Infiltrating immune cells were largely excluded from the analysis by setting the nucleus and cell parameters accordingly.

### Western blot

Cells were lysed in Pierce RIPA buffer (Thermo Scientific) containing complete EDTA-free protease inhibitor and PhosSTOP (both Roche). For extraction from tumors, the tumor tissue was minced and homogenized by sequentially filtering through 100 µm and a 70 µm nylon cell strainers with PBS on ice and then pelleted by centrifugation. Protein lysates were cleared by centrifugation at 14,000 g for 15 min. Protein content was determined using the Pierce BCA Protein kit (Thermo Fisher) according to the manufacturer’s protocol. Protein lysates were separated on NuPAGE 10% Bis-Tris-Gels (Thermo Fisher) and transferred onto a nitrocellulose blotting membrane (GE Healthcare). Membranes were blocked for 1 hour in blocking reagent (5% skim milk) in TBST (tris-buffered saline and Tween 20) and incubated with primary antibodies in blocking reagent overnight at 4°C. A list of all antibodies used for western blot can be found in the Online supplemental material and methods section. Secondary antibodies were incubated for 1 hour at room temperature and signals were detected using SuperSignal West Pico PLUS Chemiluminescent Substrate (Thermo Fisher) on a Fusion FX imaging system. For some figures, data from two independent western blots are presented together within one figure to increase ease of readability. Raw blots of all presented western blot data are provided in online supplemental figure S1.

### Gene expression analysis by quantitative real-time PCR

Total RNA was extracted from cell pellets using the innuPREP RNA mini Kit 2.0 (Analytik Jena). For extraction from B16.OVA tumors, the tumor tissue was minced and homogenized by sequentially filtering through 100 µm and 70 µm nylon cell strainers with PBS on ice and then pelleted. RNA concentrations were measured using a NanoDrop Spherospectrometer. Complementary DNA (cDNA) was reversely transcribed using the Maxima H Minus cDNA Synthesis Kit (Thermo Fisher). 1–10 ng of cDNA were analyzed by quantitative PCR using PowerUP SYBR Green (Thermo Fisher) in a QuantStudio5 Real Time PCR System. The specific primer pair sequences are listed in the Online supplemental material and methods section. The relative transcript level of each gene was normalized to *Actb* (β-Actin).

### RNAseq analysis of primary human melanoma samples

The melanoma patient cohort undergoing anti-PD-1 ± anti-CTLA-4 treatment has been described previously.^9^ Pretreatment RNA sequencing (RNA-Seq) data was available for analysis from 41 patients treated with anti-PD-1 monotherapy and 32 patients treated with combination therapy. RNA-Seq data and clinical data from The Cancer Genome Atlas (TCGA) were downloaded for 472 cutaneous melanoma samples from cBioPortal^10^ (study ID “skcm_tcga”) using the R package cgds^11^ and GENI.^12^ The vast majority of included tumor samples were reported as *cutaneous melanoma, not otherwise specified* (414). A small number of other histological subtypes were included in the analyzed TCGA dataset: *nodular* (20), *epithelioid* (8), *amelanotic* (7), *superficial spreading* (4), *desmoplastic* (3), *acral-lentiginous* (2), *lentigo maligna* (2) and *spindle cell* (2). Excluding patients with missing data for survival or *MLKL* expression resulted in 458 patients for analysis. For the multivariate Cox regression analysis, which included Union for International Cancer Control (UICC) stage as a covariate, complete data was available for 424 patients.

### Cytokine & chemokine profiling

Murine tumors were strained twice through 70 µm and 100 µm nylon strainers (BD Bioscience) using PBS with protease inhibitors (Roche). Tumor tissue from three individual mice per group was pooled to attain one sample. Triton X-100 (Sigma) was added to a final concentration of 1% and samples were frozen at −80°C, thawed again, and centrifuged at 10,000×g for 5 min. Protein content was quantified using the Pierce BCA Protein kit (Thermo Fisher) according to the manufacturer’s protocol. 200 µg protein per sample were used for downstream analysis of cytokines and/or chemokines using the Proteome Profiler Mouse Cytokine Array Kit, Panel A (R&D Systems) following the manufacturer’s protocol.

### Statistics

All data are presented as mean±SEM. Statistical significance of single experimental findings was assessed with the independent two-tailed Student’s t-test or Mann-Whitney test for data that did not meet normality criteria. For tumor growth, the pairwise intergroup comparison was calculated using the tumor growth tool from the Kroemer Lab (https://kroemerlab.shinyapps.io/TumGrowth/) for longitudinal analysis using mixed-effect modeling with Bonferroni p value adjustment and log transformation.^13^ Overall survival was analyzed using the Log-rank (Mantel-Cox) test. Significance was set at p values<0.05, p<0.01, p<0.001 and p<0.0001 and was then indicated with an asterisk (*, **, *** and ****) or as not significant. Apart from tumor growth analysis, all statistical calculations were performed using Prism (GraphPad Software).

## Results

### Loss of tumor-intrinsic MLKL impairs efficacy of cancer immunotherapy with checkpoint inhibitors

**Figure 1:**
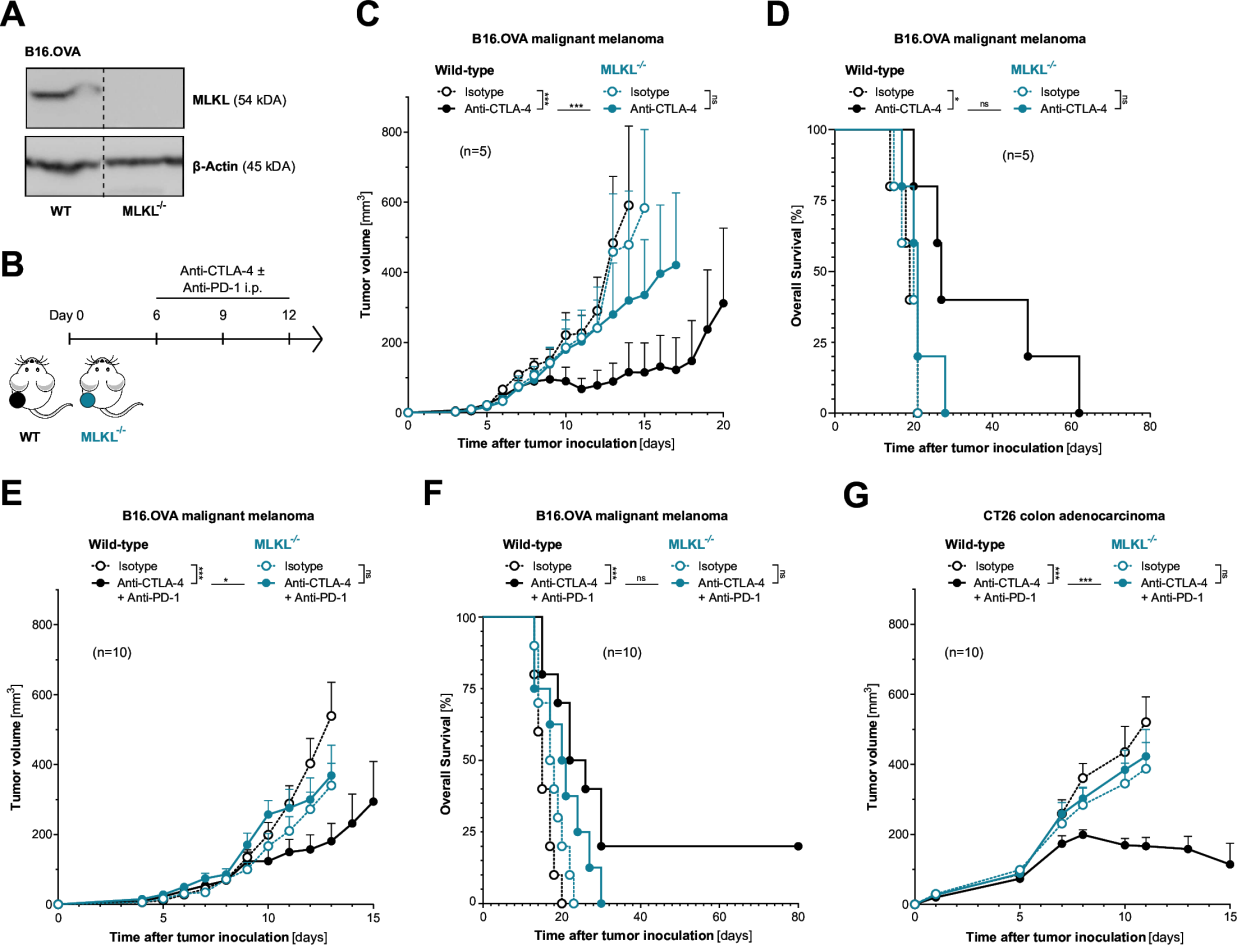
Loss of tumor-intrinsic MLKL impairs the efficacy of cancer immunotherapy with immune checkpoint inhibitors**.** (**A**) MLKL protein expression in gene-engineered cell lines was assessed using western blotting. (**B**) Treatment scheme: Mice were inoculated with either wild-type (WT) or MLKL-deficient (MLKL^−/−^) tumor cells and were injected intraperitoneally with anti-CTLA-4 ± anti-PD-1 or isotype control antibodies. (**C**) Tumor growth and (**D**) overall survival of C57BL6/J mice bearing either WT or MLKL^−/−^ B16 melanoma tumors after treatment with anti-CTLA-4. (**E**) Tumor growth and (**F**) overall survival of mice bearing either WT or MLKL^−/−^ B16 melanoma tumors after treatment with anti-CTLA-4 in combination with anti-PD-1. (**G**) Tumor growth in BALB/c mice inoculated with either WT or MLKL^−/−^ CT26 colon adenocarcinomas after treatment with anti-CTLA-4 and anti-PD-1. Data show mean tumor volume±SEM or survival for n=5–10 mice per group that are either pooled from or representative of two independent experiments. CTLA-4, cytotoxic T-lymphocytes-associated protein 4; i.p., intraperitoneal; MLKL, mixed lineage kinase domain-like pseudokinase; OVA, ovalbumin; PD-1, programmed cell death protein-1.

To address the role of tumor cell necroptosis in cancer immunotherapy with ICI, we used CRISPR-mediated somatic mutagenesis to generate polyclonal tumor cell lines that lack critical components of the necroptotic cell death molecular machinery (RIPK3^−/−^, MLKL^−/−^). C57BL/6J mice bearing either wild-type or necroptosis-deficient B16 melanomas expressing the model antigen ovalbumin (OVA) were treated with murine equivalents of clinically approved ICI of CTLA-4±PD-1 (figure 1A–B). Therapeutic inhibition of CTLA-4, which aims to invigorate de novo priming and expansion of tumor-specific T cells was indeed less efficient in mice bearing MLKL^−/−^ melanomas. This was shown by reduced tumor control and poor overall host survival when compared with wild-type tumor-bearing mice (figure 1C–D). Also, for the clinically more relevant combination of anti-PD-1 and anti-CTLA-4, we found strongly reduced antitumor activity in mice bearing melanoma with genetic deficiency for MLKL (figure 1E–F). We did not observe significantly more aggressive tumor growth and associated reduced host survival in animals bearing MLKL^−/−^ tumors in the absence of ICI treatment. These findings suggest that with the murine tumor models used here, even with functional MLKL, tumor immunogenicity is not sufficient to induce relevant tumor immunosurveillance in a steady state without additional ICI.

The dependency on tumor cell-intrinsic activity of the necroptosis executioner MLKL for ICI immunotherapy was found across different mouse strains and tumor models. MLKL deficiency in CT26 colon adenocarcinoma (without expression of an artificial antigen such as OVA) also resulted in strongly impaired antitumor activity of combined anti-PD-1/CTLA-4 ICI immunotherapy in tumor-bearing BALB/c mice (figure 1G and online supplemental figure S2A–B). Interestingly, in the murine pancreatic adenocarcinoma model Panc02.OVA with high susceptibility to ICI, tumor-intrinsic MLKL deficiency did not impact anti-CTLA-4-mediated tumor control and had only minor negative effects on tumor-bearing mice undergoing anti-PD-1 monotherapy (online supplemental figure S2C–E). Taken together, these data suggest that for some tumors intrinsic defects in MLKL can result in tumor resistance to ICI immunotherapy with anti-PD-1 and/or anti-CTLA-4.

### Defects in the RIPK3/MLKL necroptosis machinery render tumors resistant to ICI

**Figure 2:**
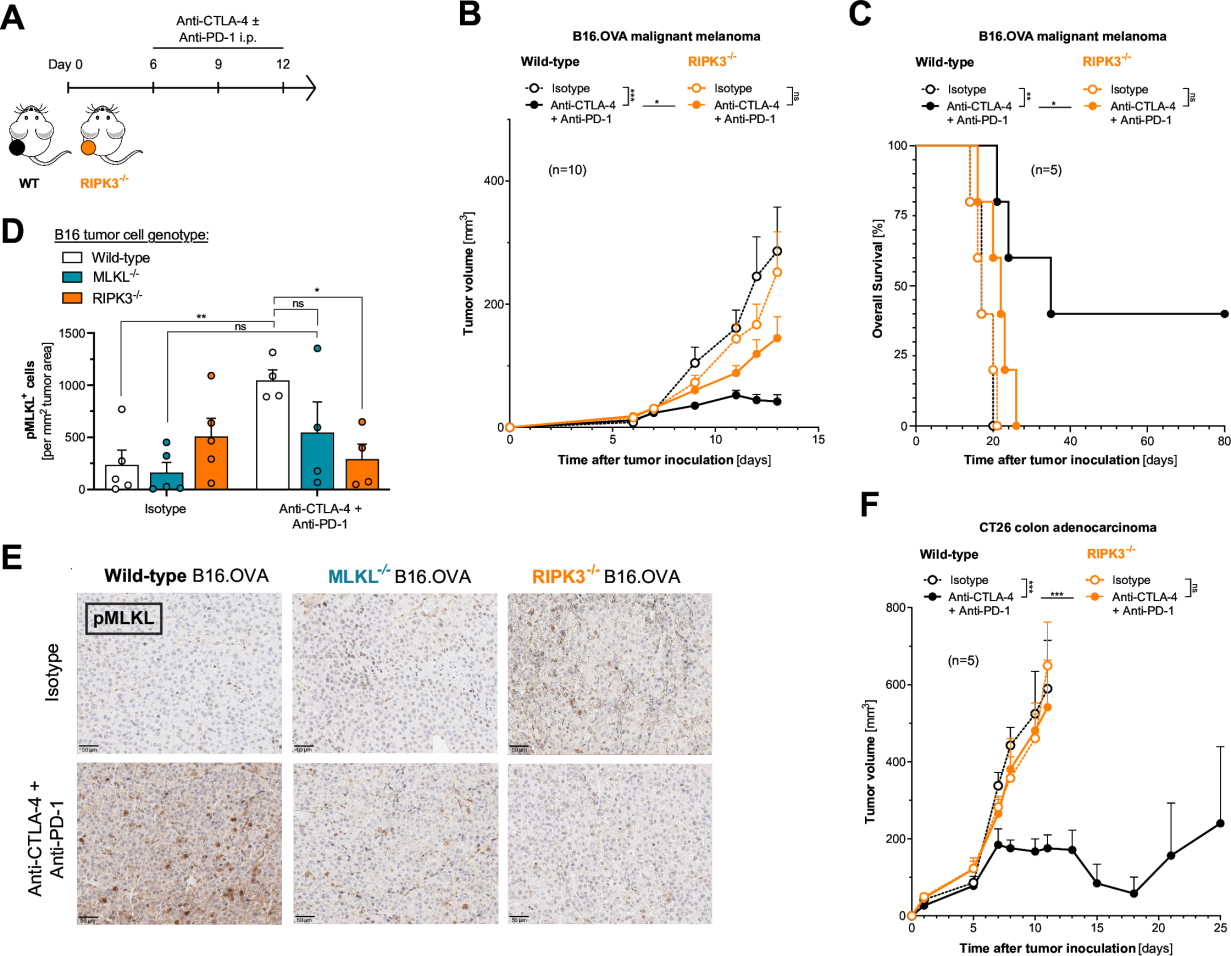
Defects in the RIPK3/MLKL necroptosis machinery render tumors resistant to ICI. (**A**) Treatment scheme: mice were inoculated with either WT, MLKL^−/−^ or RIPK3-deficient (RIPK3^−/−^) tumor cells and were injected intraperitoneally with anti-CTLA-4 and anti-PD-1 or isotype control antibodies. (**B**) Tumor growth and (**C**) overall survival of C57BL6/J mice bearing either WT or RIPK3^−/−^ B16 melanoma tumors after treatment with anti-CTLA-4 and anti-PD-1. (**D–E**) Some animals were sacrificed on day 14 and abundance of intratumoral phospho-MLKL^+^ (pMLKL^+^) cells was determined by immunohistochemistry (IHC). (**D**) Graphed data give mean pMLK^+^ cells ± SEM from n=4–5 individual mice per group. Data are representative of two independent experiments. (**E**) Representative microscopy images. (**F**) Tumor growth in BALB/c mice inoculated with either WT or RIPK3^−/−^ CT26 colon adenocarcinomas after treatment with anti-CTLA-4 and anti-PD-1 on days 5, 8, and 11. Data show mean tumor volume±SEM or survival for n=5–10 mice per group that are either pooled from or representative of two independent experiments. CTLA-4, cytotoxic T-lymphocytes-associated protein 4; ICI, immune checkpoint inhibitor; i.p., intraperitoneal; MLKL, mixed lineage kinase domain-like pseudokinase; OVA, ovalbumin; PD-1, programmed cell death protein-1; pMLKL, phosphorylated MLKL; RIPK3, receptor interacting protein kinase 3; WT, wild type.

Previous studies have suggested additional non-necroptosis-associated functions of MLKL independent of its phosphorylation by RIPK3.^14^ Therefore, we next addressed whether cancer immunotherapy with ICI also relies on the activity of RIPK3 as a component of the necroptosis pathway upstream of MLKL. Indeed, we found that loss of RIPK3 in melanoma cells phenocopied the ICI immunotherapy resistance of MLKL-deficient tumors. C57BL/6 J mice bearing RIPK3^−/−^ melanomas showed diminished tumor control and poor survival when undergoing combined therapy with anti-PD-1 and anti-CTLA-4 (figure 2A–C and online supplemental figure S3A). Along the same lines, we found particularly high intratumoral signals of pMLKL—as a surrogate marker for activation of the terminal necroptosis pathway leading to necroptotic cell death—upon ICI treatment in mice bearing wildtype but not MLKL^−/−^ or RIPK3^−/−^ tumors (figure 2D–E). Of note, the indicated genetic deficiencies were only present in tumor cells while tumor-infiltrating immune or stroma cells in the TME were proficient for RIPK3 and MLKL in these experiments. Similarly impaired antitumor activity of combination ICI was also found in BALB/c mice bearing RIPK3^−/−^ CT26 colon carcinoma tumors (figure 2F and online supplemental figure S3B–C). In contrast, in the highly ICI-susceptible model Panc02.OVA, tumor-intrinsic deficiency for RIPK3 was not associated with significant loss of tumor control in C57BL6/J host mice (online supplemental figure S3D–F). Taken together, defects in individual components of the necroptosis pathway resulted in comparable tumor resistance patterns to ICI therapy. These data suggest that this form of immunotherapy indeed relies on active necroptosis signaling in cancer cells rather than individual non-canonical functions of either RIPK3 or MLKL.

### Defects in the MLKL necroptosis machinery result in reduced cDC1 function and impaired cross-priming of tumor antigen-specific T cells

**Figure 3:**
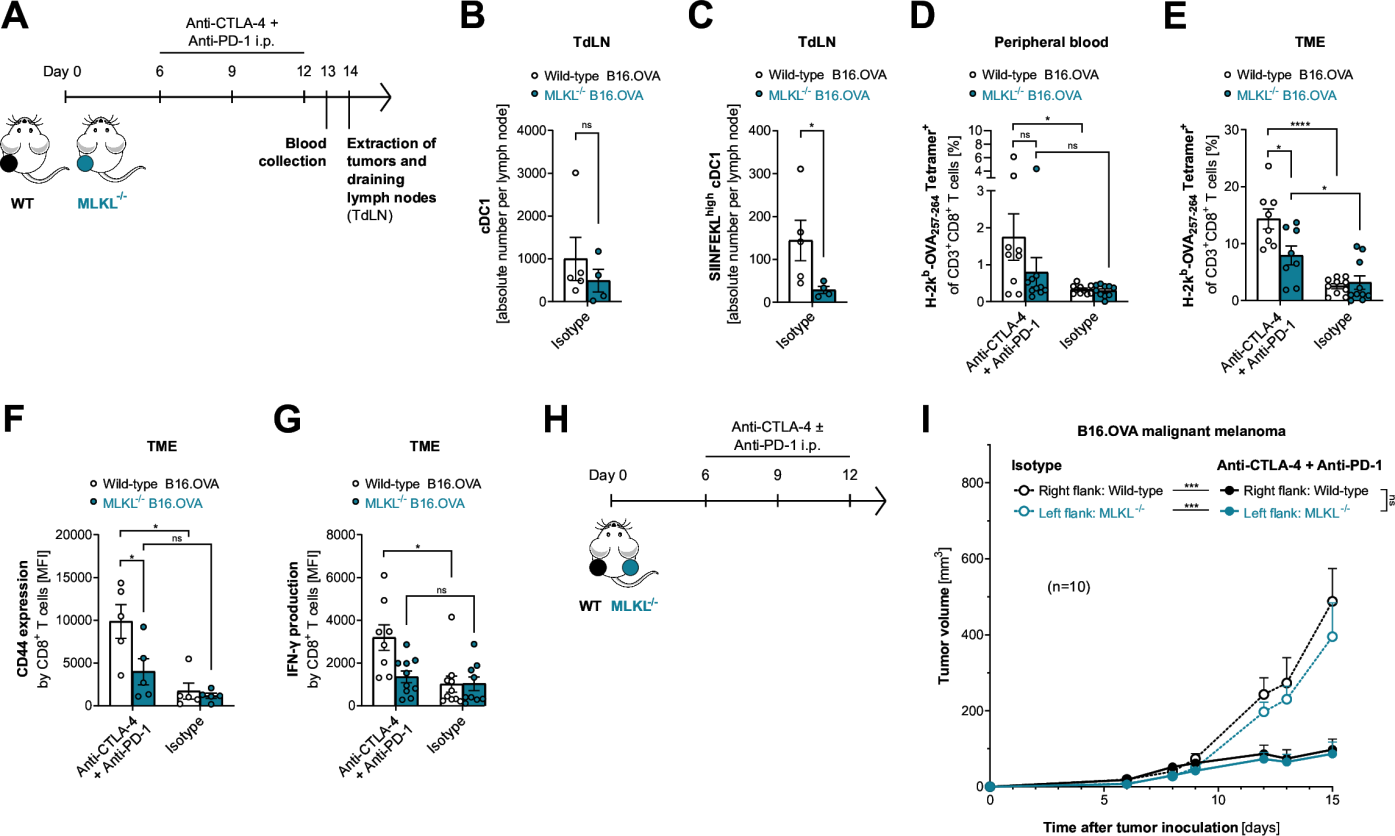
Defects in the MLKL necroptosis machinery result in reduced cDC1 function and impaired cross-priming of tumor antigen-specific T cells. (**A**) Experimental setup 1: mice injected with either WT or MLKL^−/−^ B16.OVA melanoma cells were treated with anti-CTLA-4 and anti-PD-1 or isotype control i.p. on days 6, 9, and 12. Blood was sampled on day 13, tumors and draining lymph nodes (TdLN) were extracted on day 14 and analyzed by flow cytometry. (**B**) Absolute count of cDC1 and (**C**) MHC-I SIINFEKL^high^ cDC1 in TdLNs. cCD1 were defined as CD11c^+^ CD11b^−^ CD103^+^ CD8^+^ MHC-II^+^ cells. (**D**) Frequency of H-2Kb-SIINFEKL Tetramer^+^ CD8^+^ T cells in the peripheral blood and (**E**) in the tumor microenvironment (TME). Expression of (**F**) CD44 and (**G**) IFNγ in CD8^+^ T cells in the TME presented as mean fluorescence intensity (MFI). (**H**) Experimental setup 2: mice were injected in a bilateral B16.OVA melanoma tumor model with WT cells on the right flank and MLKL^−/−^ cells on the left flank. Mice were then treated with either anti-CTLA-4 and anti-PD-1 or isotype control antibodies. (**I**) Tumor growth in the bilateral tumor model. All data are shown as mean values±SEM for n=5–10 individual mice per group that are either pooled from or representative of two independent experiments. cDC1, conventional type 1 dendritic cell; CTLA-4, cytotoxic T-lymphocytes-associated protein 4; IFNγ, interferon-gamma; i.p., intraperitoneal; MHC-I, major histocompatibility complex-I; MLKL, mixed lineage kinase domain-like pseudokinase; ns, not significant; OVA, ovalbumin; PD-1, programmed cell death protein-1; WT, wild type.

cDC1s have been associated with cancer patient survival and, in preclinical models, were found to be critical for the spontaneous rejection of immunogenic cancers and for the success of T cell-based immunotherapies.^15^ Their unique role is reflected in part by their ability to initiate de novo T-cell responses after migrating to tumor-draining lymph nodes, as well as to cross-present tumor antigens within the TME, enhancing local cytotoxic T-cell function. Therefore, we assessed whether defects in MLKL-mediated necroptosis in tumor cells impacted DC functionality and the formation of ICI-enhanced antitumor T-cell responses, using our established model of MLKL^−/−^ B16 melanoma-bearing mice (figure 3A). In adjacent tumor-draining lymph nodes of MLKL^−/−^ melanomas, we found reduced abundance of cDC1s compared with wild-type tumors (figure 3B and online supplemental figure S4A). We then analyzed the processing of the tumor-associated antigen OVA and cross-presentation of its immune-dominant peptide epitope SIINFEKL in the context of MHC class I by cDC1s. We found that cross-presentation of tumor-associated antigen by cDC1s in draining lymph nodes of MLKL^−/−^ tumors was significantly reduced compared with mice bearing wild-type tumors (figure 3C).

Reduced cross-presentation associated with MLKL^−/−^ tumors resulted in impaired cross-priming and ICI-mediated expansion of tumor antigen-specific cytotoxic T cells. While combined ICI immunotherapy in mice bearing wild-type melanomas potently enhanced the frequency of circulating tumor model antigen OVA-specific CD8^+^ T cells, this therapy-induced beneficial effect was largely abrogated in mice bearing MLKL^−/−^ tumors (figure 3D). In line with that, ICI-induced accumulation of tumor antigen-specific CD8^+^ T cells in the TME was strongly reduced in MLKL-defective tumors (figure 3E). In mice bearing melanomas without functional MLKL-mediated necroptosis signaling, ICI immunotherapy failed to boost activation and cytolytic function of CD8^+^ tumor-infiltrating T cells (figure 3F–G) and had reduced effects on CD4^+^ T cell activation (online supplemental figure S4B). Taken together, these data show that defective MLKL function in tumor cells resulted in reduced tumor antigen cross-presentation by cDC1s and suboptimal expansion and cytolytic function of tumor-infiltrating T cells upon ICI therapy.

To distinguish whether disruption of MLKL-mediated tumor cell necroptosis only affected cross-priming of CD8^+^ T cells or whether it intrinsically impaired tumor cell susceptibility to cytolytic T cell-mediated killing, we established a model in which mice were inoculated with a wild-type tumor in one flank and a MLKL^−/−^ tumor in the other flank (figure 3H). Upon combined ICI immunotherapy treatment of mice bearing chimeric tumors, we observed similar tumor growth control of both wild-type and MLKL^−/−^ melanomas (figure 3I). These data suggest that locally restricted tumor-intrinsic activity of MLKL is sufficient to mount a systemic antitumor T cell response. Once activated and expanded—presumably in lymph nodes draining tumors with active MLKL-mediated necroptosis—CD8^+^ T cells are readily able to kill MLKL^−/−^ tumor cells.

### RIPK3/MLKL-mediated tumor cell necroptosis fosters uptake of tumor cell debris by and maturation of bystander APCs

**Figure 4:**
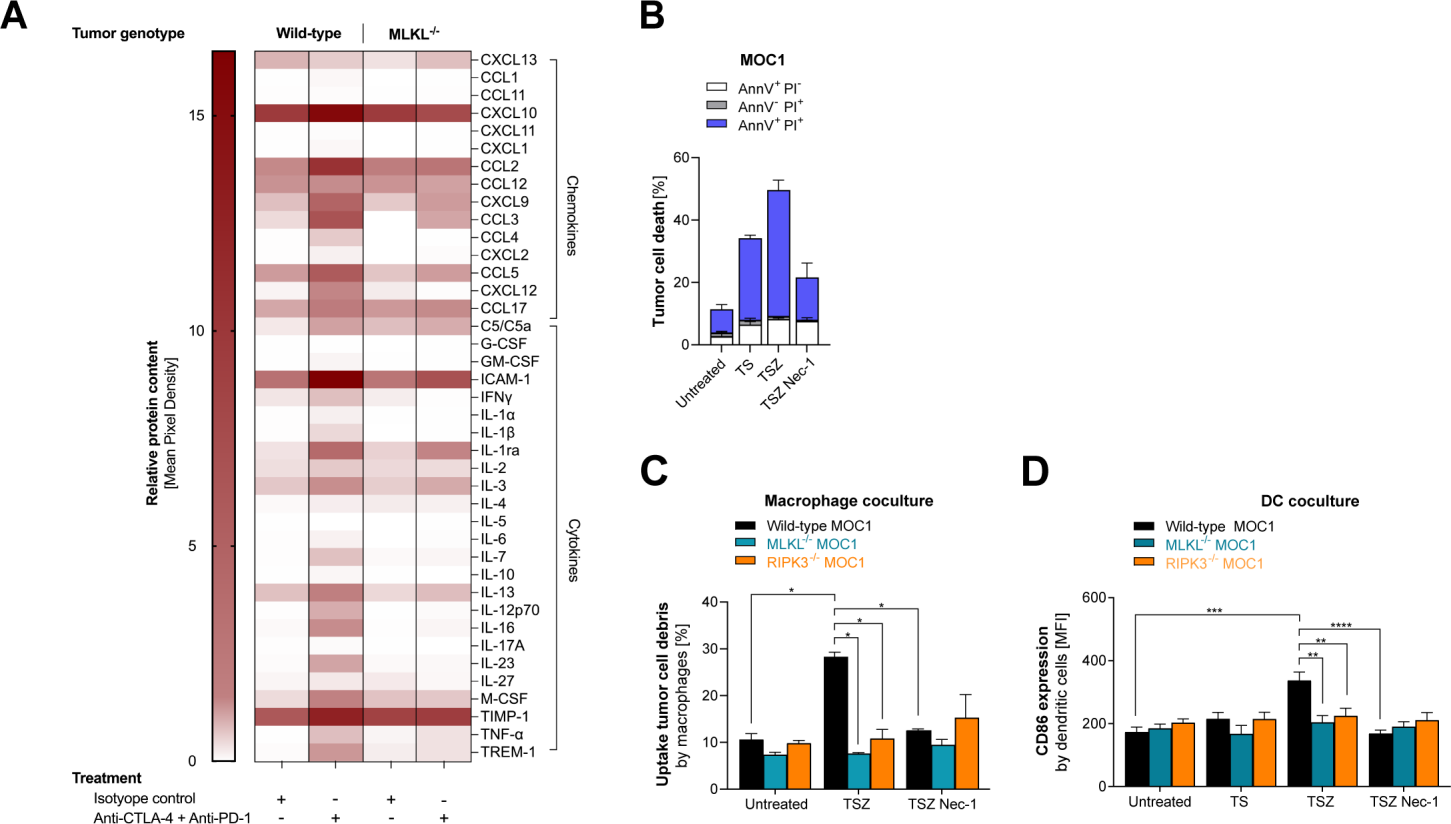
RIPK3/MLKL-mediated tumor cell necroptosis fosters uptake of tumor cell debris by and maturation of bystander APCs. (**A**) Mice inoculated with either WT or MLKL^–/–^ B16.OVA melanoma cells were treated with anti-CTLA-4 and anti-PD-1 or isotype control i.p. on days 6, 9, and 12 as described for figure 3. Tumor tissue was extracted on day 14 and was pooled from three individual mice per group for subsequent ex vivo cytokine and chemokine profiling. Heatmap showing the relative abundance of cytokines and chemokines in the TME. (**B**) Regulated tumor cell death in MOC1 oral squamous carcinoma cells after exposure to TNF-α and a SMAC-mimetic (TS, apoptosis induction), combination of TS and the pan-caspase inhibitor zVAD (TSZ, necroptosis induction), or additional necrostatin-1 (TSZ Nec-1) to inhibit necroptosis. (**C–D**) Bone marrow-derived DCs or macrophages were co-cultured with either WT, MLKL^–/–^ or RIPK3^–/–^ CFSE-labeled MOC1 tumor cells undergoing different forms of cell death and were then analyzed by flow cytometry. (**C**) Uptake of CFSE^+^ tumor cell debris by macrophages (defined as CD11b^+^ F480^+^). (**D**) CD86 expression on dendritic cells (defined as CD11c^+^) after co-culture. APCs, antigen-presenting cells; CTLA-4, cytotoxic T-lymphocytes-associated protein 4; DC, dendritic cell; GM-CSF, granulocyte-macrophage colony-stimulating factor; IFNγ, interferon-gamma; IL, interleukin; i.p., intraperitoneal; MLKL, mixed lineage kinase domain-like pseudokinase; OVA, ovalbumin; PD-1, programmed cell death protein-1; RIPK3, receptor interacting protein kinase 3; TME, tumor microenvironment; WT, wild type.

To identify factors that may contribute to the immunogenicity of MLKL-driven local necroptotic tumor cell death, we performed a broad cytokine and chemokine screening within the TME of either wild-type or MLKL^−/−^ tumors in mice undergoing checkpoint inhibitor immunotherapy. Here, we expectedly identified an array of chemokines and cytokines induced in the TME during anti-CTLA-4 + anti-PD-1 checkpoint inhibitor treatment (figure 4A). Among the factors for which induction was most prominently abolished in mice bearing MLKL^−/−^ tumors were the chemokines CCL2, CCL3, CCL5 and CXCL9. Some of these chemokines have previously been linked to the recruitment of APCs into tumors and subsequent cross-priming of tumor-reactive T cells. CCL5 has been shown to be critical for the migration of cDC1s into tumors, where they are essential for antigen cross-presentation and are also a major source of CXCL9.^16 17^ CXCL9, in turn, can act as a potent chemoattractant for activated T cells.^18^ These data suggest that necroptotic tumor cell death may initiate or amplify a cascade of events that results in chemokine-mediated recruitment of immune cells into the TME to drive ICI-enhanced antitumor immunity.

For further in vitro characterization of APC/tumor cell interactions, we induced proapoptotic signaling in tumor cells by exposure to recombinant TNF-α in combination with a small molecule SMAC (second mitochondrial-derived activator of caspases) mimetic, followed by the pan-caspase inhibitor zVAD (collectively referred to as TSZ) to skew cell death signaling towards necroptosis. However, in vitro necroptosis assays are limited by the fact that many cancer cell lines show epigenetic silencing of *RIPK3* under artificial in vitro culture conditions.^19^ Indeed, while tumor cell lysates isolated from in vivo growing B16 melanomas showed strong RIPK3 expression, short-term tumor cell culture and in particular prolonged in vitro passaging resulted in rapid loss of RIPK3 but not MLKL protein expression (online supplemental figure S5A). In line with that, B16 melanoma cells were largely resistant to in vitro induction of necroptosis, while Panc02 pancreatic and CT26 colon adenocarcinoma showed some in vitro necroptosis susceptibility (online supplemental figure S5B).

The murine oral squamous cell carcinoma cell line MOC1 showed particularly high in vivo responsiveness to ICI immunotherapy, which similarly to B16 melanoma was completely dependent on tumor cell-intrinsic MLKL activity (online supplemental figure S5C). However, in contrast to B16 melanoma, MOC1 cells showed consistently strong RIPK3 expression levels during in vitro culture (online supplemental figure S5D). In line with that, MOC1 carcinoma cells were highly susceptible to necroptosis with rapid cell death induction on exposure to TSZ, which was completely blocked by the necroptosis/RIPK1 inhibitor necrostatin-1 (figure 4B). Upon co-culture with fluorescently labeled MOC1 cells undergoing TSZ-induced necroptosis, BMDMs showed increased uptake of carcinoma cell debris, which was completely absent with RIPK3^–/–^ or MLKL^–/–^ carcinoma cells (figure 4C). In BMDCs, we found upregulation of the costimulatory molecule CD86 upon interaction with necroptotic MOC1 cells which was dependent on active RIPK3 and MLKL in carcinoma cells and was blocked by necrostatin-1 (figure 4D). Taken together, these data demonstrate that tumor cells undergoing RIPK3/MLKL-mediated necroptotic cell death can induce uptake of tumor debris by and maturation of bystander APCs.

### Epigenetic upregulation of the necroptosis machinery by hypomethylating agents can augment tumor susceptibility to ICI immunotherapy

Apart from the above-mentioned artificial loss of RIPK3 expression under in vitro culture conditions, epigenetic silencing by hypermethylation of promoter regions of critical necroptosis machinery components, particularly RIPK3, has been identified as a common escape mechanism in different cancer types in vivo.^19 20^ Hypomethylating agents such as 5-AD or 5-AZA have been used in experimental settings to circumvent epigenetic silencing of RIPK3 and MLKL. In contrast to its immunogenic counterpart MOC1, the poorly immunogenic carcinoma cell line MOC2 showed little expression of RIPK3 and consequently no susceptibility to TSZ-induced necroptotic cell death in vitro (online supplemental figure S6A-B). Exposure to 5-AD upregulated the transcriptional activity of *RIPK3* and *MLKL* in MOC2, rendering the cell line susceptible to TSZ-induced necroptosis in vitro (online supplemental figure S6B-C).

**Figure 5:**
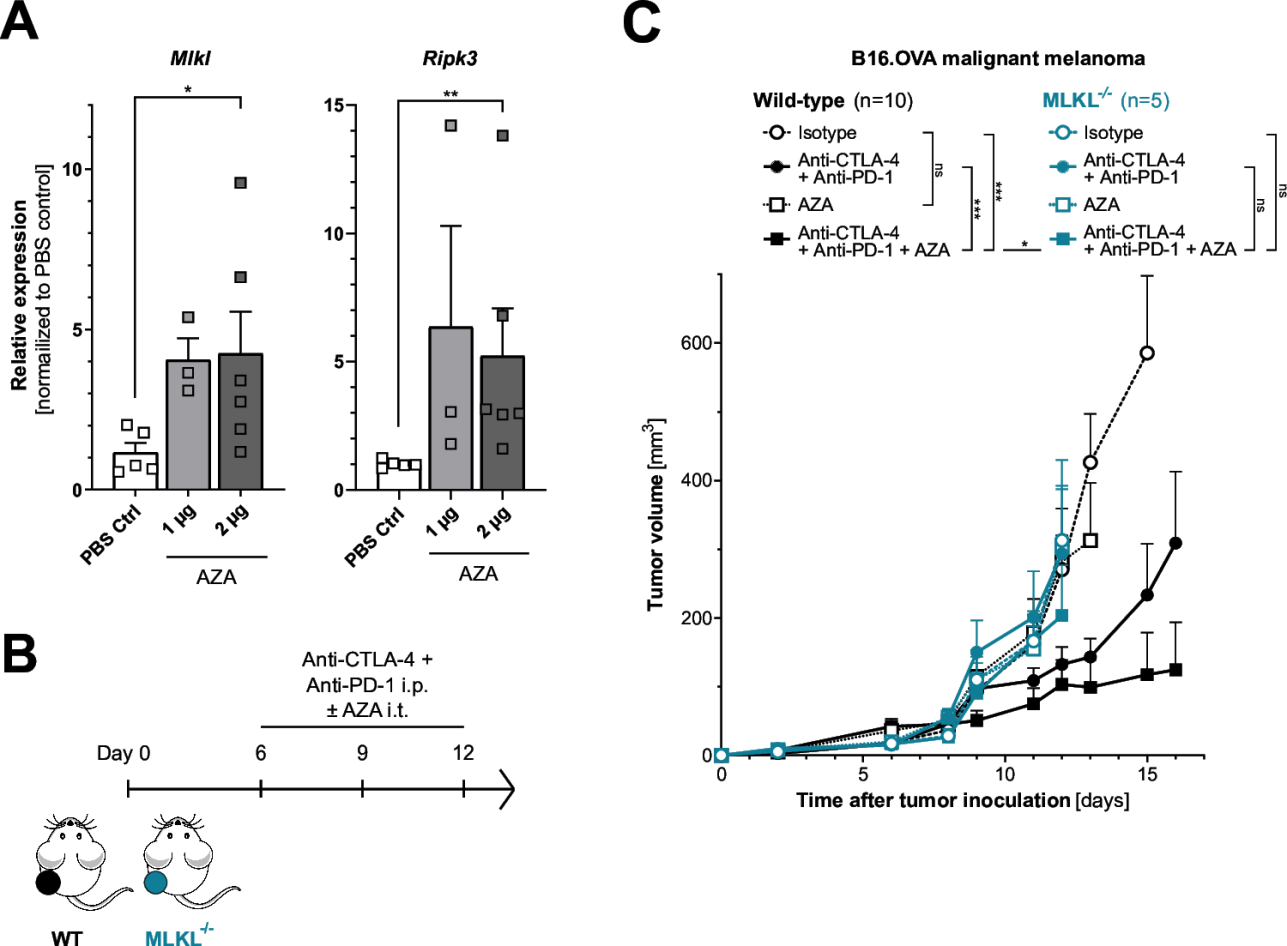
Epigenetic upregulation of the necroptosis machinery by hypomethylating agents can augment tumor susceptibility to ICI immunotherapy. Mice were inoculated with B16.OVA WT cells and treated with two injections of intratumoral 5-azacytidine (AZA) on days 8 and 11. RNA was extracted from the tumor tissue on day 12 and analyzed via qPCR. (**A**) Relative expression of *Mlkl* and *Ripk3* mRNA normalized to untreated tumors. Data were pooled from two separate experiments. (**B**) Treatment scheme: WT mice were inoculated with either WT or MLKL^−/−^ B16.OVA cells. Recipients were injected intraperitoneally with anti-CTLA-4/anti-PD-1 or isotype control antibodies in combination with intratumoral application of AZA. (**C**) Mean tumor volume±SEM of n=5–10 individual mice per group that were pooled from two independent experiments. CTLA-4, cytotoxic T-lymphocytes-associated protein 4; ICI, immune checkpoint inhibitor; i.p., intraperitoneal; i.t., intratumoral; MLKL, mixed lineage kinase domain-like pseudokinase; mRNA, messenger RNA; ns, not significant; OVA, ovalbumin; PBS, phosphate-buffered saline; PD-1, programmed cell death protein-1; qPCR, quantitative PCR; RIPK3, receptor interacting protein kinase 3; WT, wild type.

In the B16 melanoma model, intratumoral application of low-dose AZA without direct cytotoxic effects did increase transcriptional activity of both *Ripk3* and *Mlkl* in tumor cells in vivo (figure 5A and online supplemental figure S6D). When we combined intratumoral injection of low-dose AZA with systemic ICI immunotherapy, we found synergistically improved tumor control in mice bearing wild-type melanoma (figure 5B–C). In contrast, and as expected, in mice bearing tumors with irreversible genetic MLKL deficiency, epigenetic combination therapy with AZA failed to render tumors susceptible to ICI immunotherapy. Taken together, these data suggest that combination therapy with hypomethylating agents such as AZA has the potential to overcome tumor resistance to ICI immunotherapy by upregulation of otherwise epigenetically silenced components of the necroptosis pathway in tumor cells.

### High transcriptional activity of *MLKL* human melanoma correlates with prolonged survival and durable responses to ICI immunotherapy

**Figure 6:**
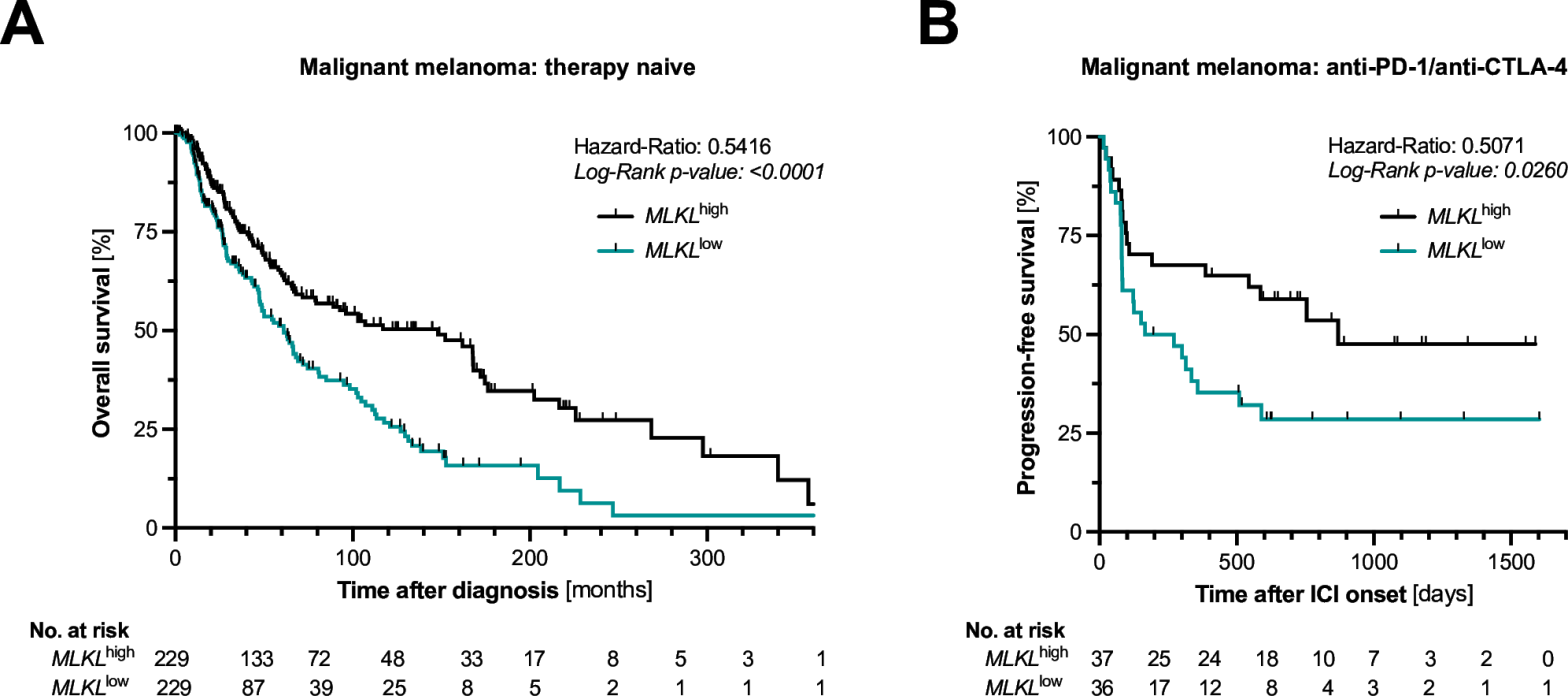
High transcriptional activity of *MLKL* in human melanoma correlates with prolonged survival and durable responses to ICI immunotherapy. (**A**) Overall survival in 458 patients with advanced malignant melanoma from TCGA by expression of *MLKL* in RNA-sequencing of bulk tumor tissue. (**B**) Progression-free survival in n=73 patients with malignant melanoma undergoing anti-PD-1±anti-CTLA-4 ICI immunotherapy by expression of *MLKL* in tumor samples. CTLA-4, cytotoxic T-lymphocytes-associated protein 4; ICI, immune checkpoint inhibitor; MLKL, mixed lineage kinase domain-like pseudokinase; PD-1, programmed cell death protein-1; TCGA, The Cancer Genome Atlas.

To explore the potential clinical relevance of our findings, we analyzed genome-wide transcriptional programs in 458 primary melanoma patient samples from TCGA determined by RNA-Seq. Using an unbiased approach, median expression of *MLKL* was used as a cut-off to classify patients into a low and high expression subgroup. In line with our preclinical data, we found that high expression of *MLKL* was associated with prolonged overall survival in patients with malignant melanoma (figure 6A). Multivariable Cox regression identified low *MLKL* expression in melanoma samples as an independent risk factor for death (HR 0.51, 95% CI 0.38 to 0.69) when controlling for stage of disease (UICC tumor status), age, and gender (online supplemental table S1). Gene set enrichment analysis of differentially expressed genes between patients with melanoma with high or low *MLKL* expression showed that many of the upregulated genes in *MLKL^high^* tumors clustered in inflammation-related pathways (online supplemental figure S7A). We next performed transcriptomic profiling of publicly available RNA-Seq data from a previously described patient cohort (n=73) with malignant melanoma undergoing ICI immunotherapy with either anti-PD-1 or its combination with anti-CTLA-4.^9^ We found evidence of superior disease control associated with prolonged progression-free survival in patients with high *MLKL* expression in pretreatment tumor samples (figure 6B). For a limited number of patients in this cohort, additional on-treatment tumor samples (typically acquired 5–14 days after onset of ICI treatment) were available. Interestingly, both *MLKL* and *RIPK3* gene expression levels in these paired biopsies were increased during ICI treatment, suggesting a potential positive feedback loop by infiltrating immune cells which might enhance necroptotic signaling in the TME (online supplemental figure S7B). Taken together, these patient data corroborate our preclinical findings that high transcriptional activity of *MLKL* in melanoma tissue is associated with prolonged overall survival and durable response to ICI immunotherapy.

## Discussion

Our findings that ICI immunotherapy relies on tumor cell-intrinsic RIPK3/MLKL signaling are in line with previous reports demonstrating that artificial activation of the necroptosis pathway can boost ICI antitumor efficacy. Forced overexpression via intratumoral injection of *Mlkl*-encoding mRNA in B16 melanoma or CT26 colon carcinoma resulted in IFN-I-dependent cross-priming of CD8^+^ T cells by migratory DCs and subsequent tumor eradication upon ICI treatment.^21^ Injection of genetically engineered tumor cells expressing a constitutively active form of RIPK3 synergized with ICI immunotherapy for durable tumor clearance in mice.^22^ Such ectopic introduction of necroptotic cells into the TME promoted cDC1-dependent T-cell immunity based on increased tumor antigen loading by tumor-associated APCs. While these studies provided important mechanistic learnings, they are based on highly artificial modes of necroptosis induction. In contrast, our approach with genetic deletion of *Ripk3* or *Mlkl* in tumor cells suggests some extent of spontaneous and potentially continuous pro-necroptotic signaling in tumor cells as the ignition point of basal T-cell immunity and necessity for the success of subsequent ICI immunotherapy. The exact mechanisms that may perpetuate such basal pro-necroptotic signaling in tumor cells remain to be determined. Our findings in human tumor samples that *MLKL* and *RIPK3* gene expression levels were upregulated during ICI treatment suggest a potential positive feedback loop by infiltrating immune cells which might further enhance necroptotic signaling in the TME.

Generally, despite similarity in their morphological outcome, the regulated process of necroptosis is differentiated from accidental necrosis by defined molecular triggers. These are best characterized in the context of pathogenic infections and include signaling via death receptors of the TNF family, pattern recognition receptors or IFN receptors.^7^ In tumor cells, these molecular interactions are poorly understood but nutrient deprivation or attack by infiltrating immune cells has been suggested to trigger such pro-necroptotic pathways.^6 23^ Recent studies demonstrated that the detection of aberrant nucleic acids within tumor cells via innate pattern recognition receptors and subsequent IFN-I signaling are critical for the formation of antitumor T-cell immunity and the efficacy of ICI immunotherapy.^24^,^26^ Cell-intrinsic activation of both cytosolic DNA-sensing cyclic GMP-AMP synthase/stimulator of interferon genes (cGAS/STING) and RNA-sensing retinoic acid-inducible gene I/mitochondrial antiviral signaling (RIG-I/MAVS) pathways can trigger necroptosis.^27^ In line with that, genetically engineered tumors deficient in these nucleic acid receptor systems phenocopied tumor resistance to ICI with MLKL^−/−^ tumors.^24 26^ Activation of other nucleic acid sensors such as Z-DNA binding protein 1 (ZBP1) (potentially upon detection of endogenous viral elements) can similarly induce necroptosis and immunosurveillance in murine tumors.^6 28^ Genotoxic stress seems to enhance these effects through sensing of aberrantly located self RNA and DNA by intrinsic nucleic acid receptors. Coherently, radiation or chemotherapeutic agents such as oxaliplatin and anthracyclines can actively trigger necroptosis in tumor cells and their antitumor activity in vivo was found to rely on functional MLKL.^29 30^ While our retrospective data analysis in humans suggests an association between transcriptional activity of the necroptosis pathway and the efficacy of checkpoint inhibitor immunotherapy, other studies have drawn similar links to conventional chemotherapy.^31 32^ However, the exact role of nucleic acid sensors as potential upstream activators of necroptosis in tumors and their impact on conventional and immunotherapy in patients with cancer remains to be determined.

The regulation of specific necroptosis machinery components in tumor tissue is incompletely understood. In a genome-wide screen of multiple human cancers, genetic deletion by copy number variation under selective pressure was exclusively found for proapoptotic caspases (*CASP3*, *CASP9*) but not necroptosis-associated genes (*RIPK3*, *MLKL*).^23^ Even though critical necroptosis genes may not be selectively deleted or mutated, their expression may still be suppressed via epigenetic mechanisms. Methylation-dependent loss of *RIPK3* expression is a common necroptosis resistance mechanism in cancer that seems to be driven by oncogenes and occurs progressively during tumor growth.^19 33^ Epigenetic regulation of MLKL is much less common, but a recent study in a pancancer multiomics analysis of necroptosis-related regulators associated hypermethylation of *MLKL* with poor overall survival in patients with malignant melanoma.^34^ Our translational therapeutic approach to use hypomethylating agents to resensitize tumor cells to necroptotic cell death and thus subsequent ICI immunotherapy, is in line with previous reports that have used a similar strategy in the context of chemotherapy treatment.^19 20^ Our data suggest that the immunogenic effect of AZA in treating various cancers may be at least in part linked to the upregulation of the necroptotic machinery, a hypothesis that warrants further investigation. Alternative approaches to enhance necroptosis signaling in tumor cells may include manipulation of ADAR1, which was shown to mask ZBP1-driven necroptosis,^28^ or therapeutic induction of IFN-I signaling in the TME as *MLKL* and other critical components are IFN-stimulated genes.^7^

Studies on the functional outcome of necroptosis signaling in human tumors have shown conflicting results. Our findings from patients with malignant melanoma are in line with a previous meta-analysis associating low transcriptional activity of *MLKL* in tumor samples with unfavorable disease development in various forms of cancer.^35^ Similarly, low expression of *RIPK3* in tumor biopsies was linked to poor overall survival in patients with malignant melanoma.^22^ In contrast, other studies found high MLKL activity associated with poor survival in patients with breast cancer or head and neck squamous cell carcinoma.^36 37^ Some of these discrepancies might be explained by the fact that most of the mentioned studies including our own were done from bulk tumor tissue samples, in which the source and main driver (cancer vs immune or stromal cells) of these gene expression profiles is unclear. Additionally, non-canonical functions of MLKL that are independent of its phosphorylation by RIPK3 have been described. Some of them connect MLKL with processes other than necroptosis, for example, endosomal trafficking and extracellular vesicle formation,^14 38^ which we recently linked to cancer immunosurveillance. Thus, it is important to consider the tissue, stress and disease contexts in which necroptosis occurs as well as the duration or amplitude of inflammation elicited to sufficiently determine its effects in the context of cancer.

Other forms of ICD have been linked to tumor immunosurveillance and efficacy of immunotherapy. Pyroptosis is executed by members of the pore-forming protein family of gasdermins. Gasdermin E (GSDME) has been found to act as a tumor suppressor, as its genetic loss was associated with reduced antitumor T-cell immunity and poor tumor control in mice.^39^ Triggering pyroptosis in less than 15% of tumor cells via tumor-selective release of GSDMA3 with a bioorthogonal chemical system was sufficient to induce elimination of the entire mammary tumor graft in mice.^40^ Although canonically proposed as strictly segregated cellular processes, mounting evidence shows significant interactions between the components of programmed cell death pathways. Once cleaved and activated by caspase-3, GSDME can convert apoptosis programs in tumor cells into secondary inflammatory cell death in the form of pyroptosis.^41^ Mounting evidence of plasticity in these pathways has led to the conceptualization of PANoptosis, an inflammatory cell death modality that integrates components from necroptosis, apoptosis and pyroptosis.^42^ PANoptosis cannot be individually accounted for by any canonical cell death pathway alone and has been implicated in driving innate immune responses, potentially also in the context of tumor immunosurveillance.^43^ Analysis of the human cancer transcriptome suggested tumor type-specific differences in the activity of regulated cell death pathways.^42^ Currently, it remains unclear to which extent redundancy in the function of distinct cell death mechanisms during tumor immunosurveillance exists, and how they individually shape tumor immunosurveillance in a context-dependent manner (eg, tumor type or certain modalities of cancer treatment).

In this study, we have demonstrated that basal necroptotic activity is necessary for the efficacy of ICI in several cancer entities. A better understanding of the complex and potentially opposing functions of MLKL, associated necroptosis and their demarcation to other forms of regulated cell death in cancer will be needed for therapeutic exploitation of these pathways to the benefit of patients with cancer. In doing so, enhanced necroptosis signaling in cancer cells has the potential to broaden the applicability of immunotherapies such as checkpoint inhibitors to patients with otherwise resistant tumors due to a “cold” TME.

## Supplementary material

10.1136/jitc-2024-010433 online supplemental file 1

## Data Availability

All data relevant to the study are included in the article or uploaded as supplementary information.
