# Supplementary material for: Defects in the necroptosis machinery are a cancer resistance mechanism to checkpoint inhibitor immunotherapy
Source: J Immunother Cancer. 2025 May 8;13(5):e010433. doi: 10.1136/jitc-2024-010433 (PMC12067934; doi:10.1136/jitc-2024-010433)
Supplement: online supplemental file 1 [file jitc-13-5-s001.pdf]

SUPPLEMENTARY MATERIAL AND METHODS

Phospho-MLKL immunohistochemistry

IHC antibodies:

Antibody	Isotype	Clone	Vendor
pMLKL	Rabbit IgG	Monoclonal	Abcam
Anti-rat IgG, biotinylated antibody	Rabbit IgG	Polyclonal	Vector laboratories

Western blot

Western blot antibodies:

Antibody	Isotype	Clone	Vendor
Anti-rabbit IgG, HRP-linked antibody	Mouse IgG	Polyclonal	Cell Signaling
MLKL	Rabbit IgG	Polyclonal	Cell Signaling
RIPK3	Rabbit IgG	Polyclonal	ProSci
β-Actin	Rabbit IgG	13E5	Cell Signaling

CRISPR-Cas-mediated genome editing

GuideRNA target sequences:

Gene	Target sequences
Mlkl	gRNA1: 5’-GCACACGGTTTCCTAGACGC-3’<br>gRNA2: 5’-GACTTCATCAAAACGGCCCA-3’
Ripk3	gRNA1: 5’-CGGACACGAAGTCCCACTGG-3’<br>gRNA2: 5’-TGGAGAATGGCTCCCTCGCA-3’

Quantitative real-time PCR

mRNA primer sequences:

Gene	Target sequences
Mlkl	Forward: 5’-CTGAGGGAACTGCTGGATAGAG-3’<br>Reverse: 5’-CGAGGAAACTGGAGCTGCTGAT-3’
Ripk3	Forward: 5’-GAAGACACGGCACTCCTTGGTA-3’<br>Reverse: 5’-CTTGAGGCAGTAGTTCTTGGTGG-3’
Actb (β-actin)	Forward: 5’-CATTGCTGACAGGATGCAGAAGG-3’<br>Reverse: 5’-TGCTGGAAGGTGGACAGTGAGG-3’

Flow cytometry

Live/Dead Dyes:

Fluorochrome	Dye name	Vendor	Order #
AmCyan	Fixable Viability Dye eFluor™ 506	eBioscience	65-0866-18
APC-Cy7	Fixable Near-IR Dead Cell Stain	Thermo Fisher	L10119
FITC	Annexin V	Biolegend	640906
N/A	Propidium Iodide	Biolegend	421301

Fluorochrome-coupled antibodies:

Fluoro-chrome	Target (murine)	Isotype	Clone	Vendor	Order #
FITC	CD3	Rat IgG2b, κ	17A2	Biolegend	100204
PE-Cy7	CD3	Rat IgG2b, κ	17A2	Biolegend	100219
Pacific Blue	CD4	Rat IgG2b, κ	GK1.5	Biolegend	100428
APC	CD8	Rat IgG2b, κ	53-6.7	Biolegend	100712
PerCP-Cy5.5	CD8	Rat IgG2a, κ	53-6.7	Biolegend	100734
APC	CD11b	Rat IgG2a, κ	M1/70	Biolegend	101212
APC-Cy7	CD11b	Rat IgG2a, κ	M1/70	Biolegend	101226
APC	CD11c	Armenian hamster IgG	N418	eBioscience	17-0114-82
Pacific Blue	CD11c	Armenian hamster IgG	N418	eBioscience	117322
APC-Cy7	CD44	Rat IgG2a, κ	IM7	Biolegend	103028
APC-Cy7	CD45.2	Mouse (SJL) IgG2a, κ	104	Biolegend	109824
PE-Cy7	CD45.2	Mouse (SJL) IgG2a, κ	104	Biolegend	109829
PerCP-Cy5.5	CD69	Armenian Hamster IgG	H1.2F3	Biolegend	104520
PE	CD80	Armenian Hamster IgG	16-10A1	Biolegend	104708
PerCP-Cy5.5	CD86	Rat IgG2a, κ	GL-1	Biolegend	105016

PE-Cy7	CD86	Rat IgG2a, κ	GL-1	Biolegend	105014
PE	CD103	LOU/M IgG2a, κ	M290	BD BioSciences	557495
Pacific Blue	CD107a	Rat IgG2a, κ	1D4B	eBioscience	48-1071-82
PE-Cy7	F4/80	Rat IgG2a, κ	BM8	eBioscience	25-4801-82
PE-Cy7	Granzym e B	Rat IgG2a, κ	NGZB	eBioscience	25-8898-82
PE	IFN-γ	Rat IgG2a, κ	XMG1.2	Biolegend	505808
PerCP-Cy5.5	MHC-I (H2Kd)	Mouse IgG2a, κ	34-1-2S	eBioscience	46-5998-82
Pacific Blue	MHC-I (H2Kb)	Mouse IgG2a, κ	AF6-88.5.5.3	eBioscience	48-5958-82
APC	MHC-I SIINFEK L (H2Kb)	Mouse IgG1, κ	25-D1.16	Biolegend	141606
FITC	MHC-II (I-A/I-E)	Rat IgG2a, κ	M5/114.15.2	Biolegend	107606
PE	MHC-II (I-A/I-E)	Rat IgG2a, κ	M5/114.15.2	Biolegend	107607
APC	NK1.1	Mouse IgG2a, κ	PK136	Biolegend	108710

Short-term *ex vivo* culture of freshly isolated tumor cells. Primary cultures were obtained by finely mincing extracted B16.OVA tumors on ice and digestion in 1 mL of phosphate-buffered saline (PBS) supplemented with 0,5% bovine serum albumin (BSA), 0,5 M EDTA, DNase I (100U/mL) and Collagenase II (125 U/mL) for 15 minutes at 37°C in a shaking ThermoMixer at 300 rpm. The digested tumors were filtered through a 100 µm cell strainer, pelleted by centrifugation (400xg, 5 min) and resuspended in 1mL of red blood cell lysis buffer. The lysis was stopped after 1 minute by adding 9 mL of PBS. Cells were spun down again (400xg, 5 min) and then cultured in DMEM supplemented with 15% fetal calf serum (FCS) for 24 hours before cells were lysed for protein extraction.

SUPPLEMENTARY FIGURES WITH LEGENDS

A

Fig. 1A: B16.OVA

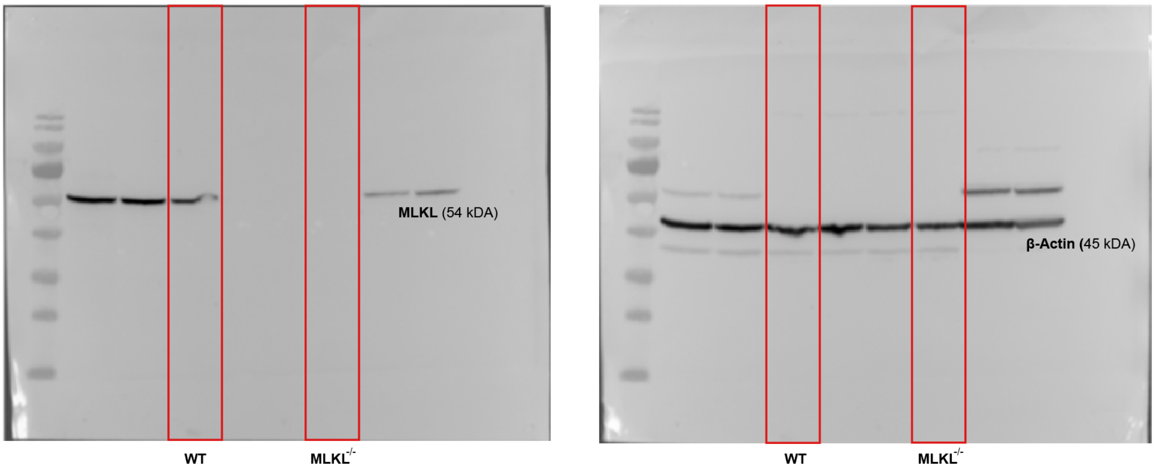

B

Fig. S2A: CT26; Fig. S2C: Panc02.OVA

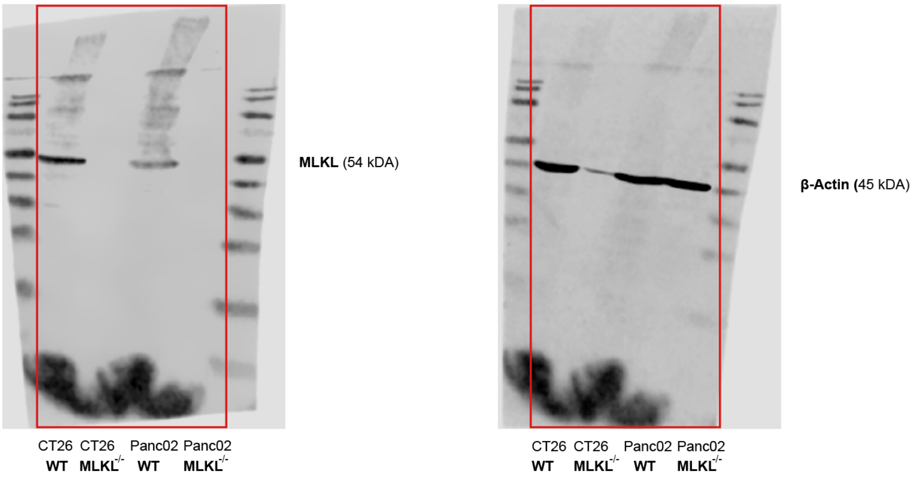

C

Fig. S3A: B16.OVA

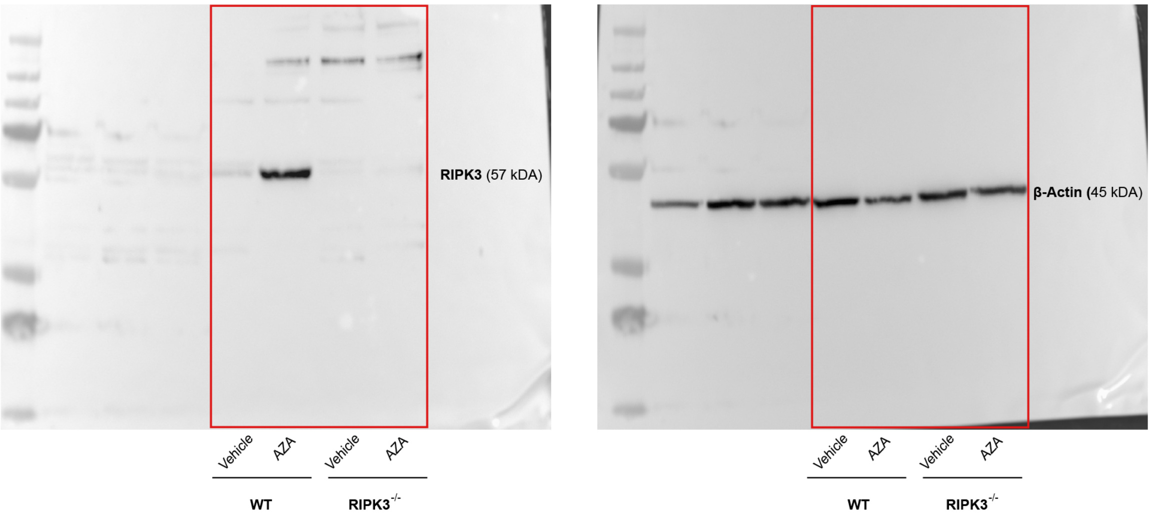

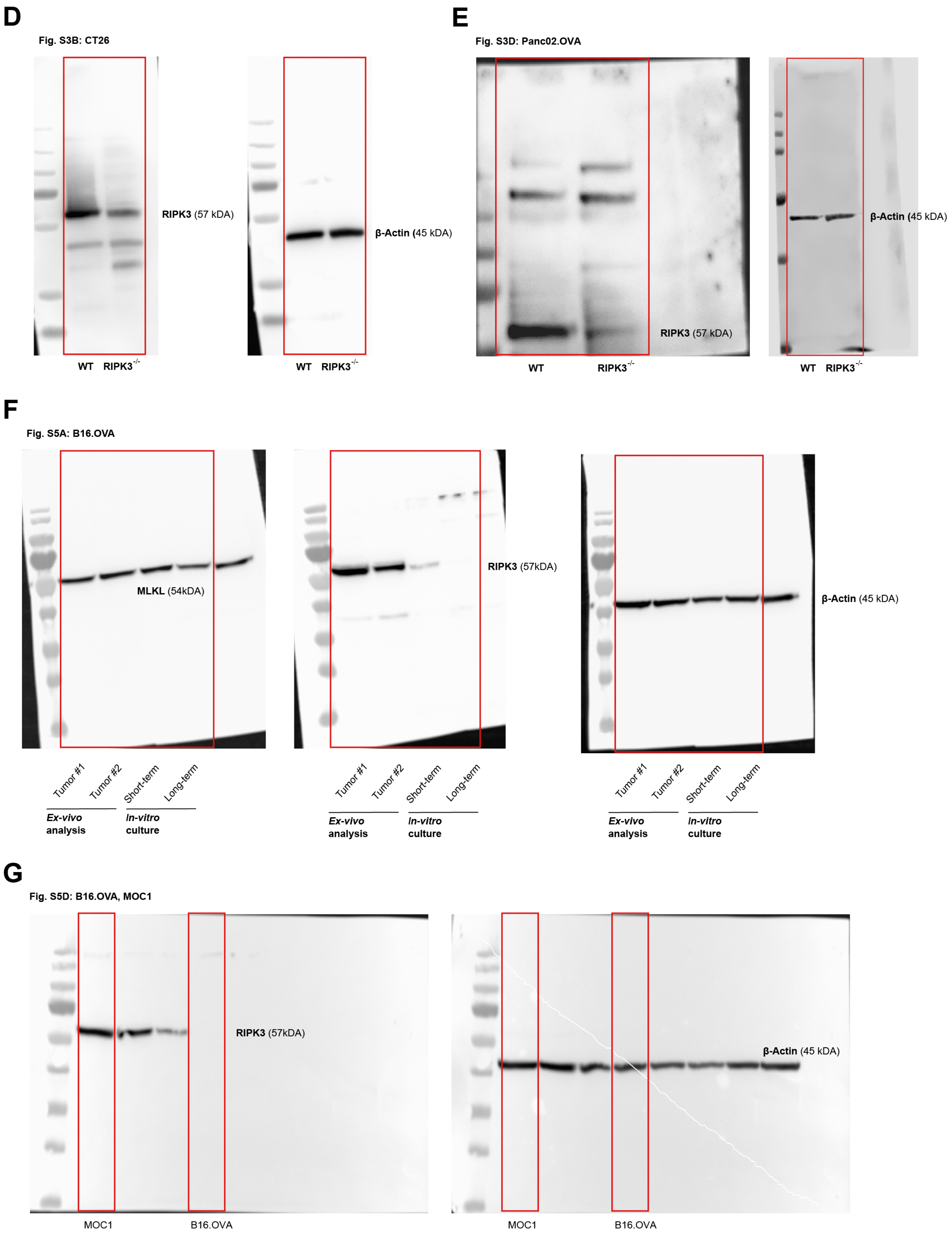

Figure S1: Raw blots of all presented western blot data. (**A**) Full blotting membrane corresponding to Fig 1A: MLKL protein expression in gene engineered B16.OVA cells. (**B**) Full blotting membrane corresponding to Fig S2A and Fig S2C: MLKL protein expression in

Panc02.OVA pancreatic adenocarcinoma cell lines. (**C**) Full blotting membrane corresponding to Fig S3A: RIPK3 protein expression in gene engineered B16.OVA cell lines. (**D**) Full blotting membrane corresponding to Fig S3B: RIPK3 protein expression in gene engineered CT26 colon adenocarcinoma cell lines. (**E**) Full blotting membrane corresponding to Fig S3D: RIPK3 protein expression in gene engineered Panc02.OVA pancreatic adenocarcinoma cell lines. (**F**) Full blotting membrane corresponding to Fig S5A: Expression of MLKL and RIPK3. *Ex vivo* analysis of two freshly isolated samples of B16.OVA tumors from C57BL/J mice (Tumor #1, Tumor #2), or analysis of B16.OVA cells after short term or long-term *in vitro* cell culture. (**G**) Full blotting membrane corresponding to Fig S5D: RIPK3 protein expression in MOC1 and B16.OVA tumor cells under *in vitro* culture conditions.

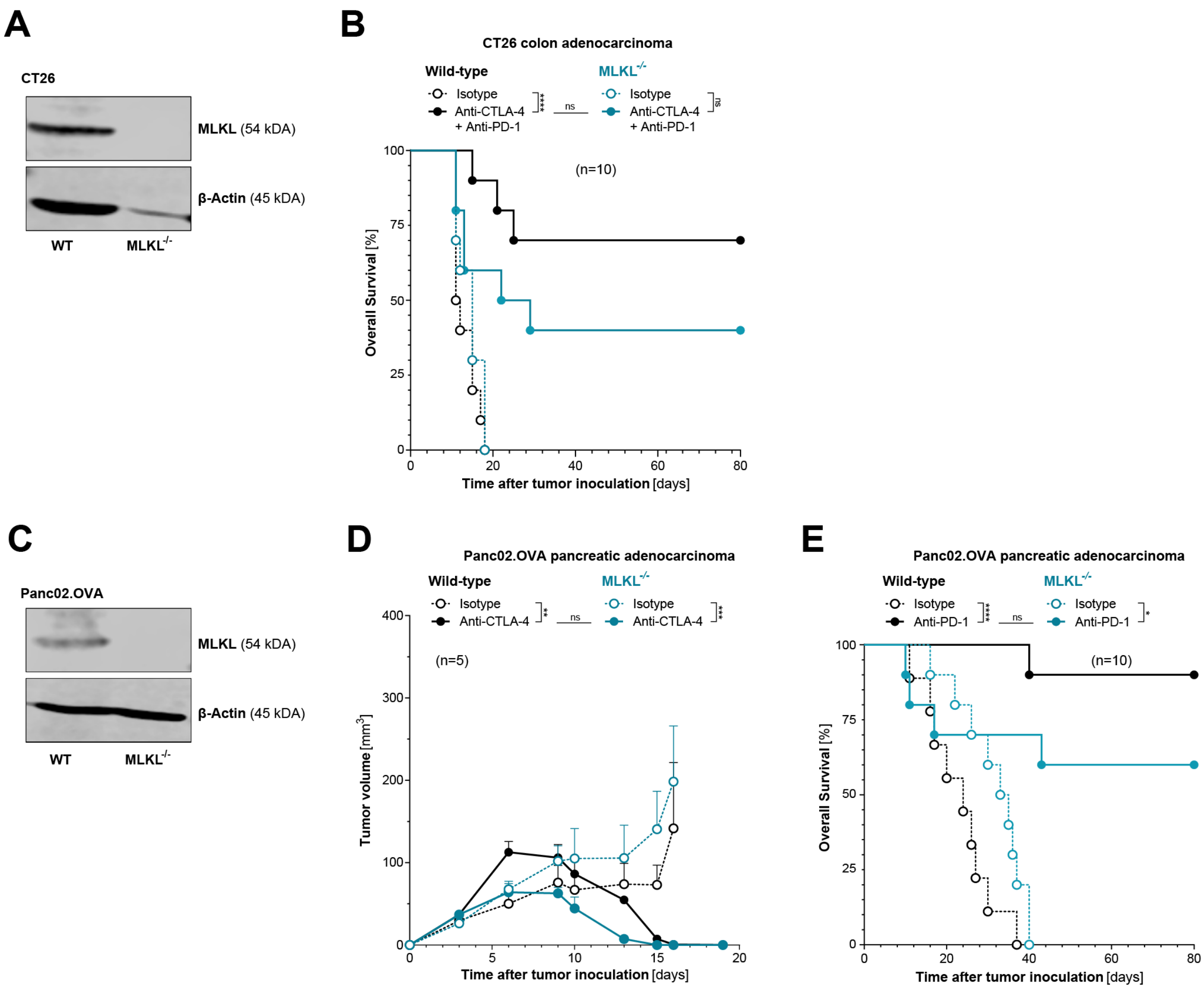

Figure S2: ICI immunotherapy in CT26 colon but not Panc02.OVA pancreatic adenocarcinoma tumors relies on intrinsic MLKL activity. (**A**) MLKL protein expression in CT26 colon adenocarcinoma cell line was assessed using western blotting. (**B**) Overall survival of n=5 BALB/c mice bearing either wild-type (WT) or MLKL-deficient (MLKL$^{-/-}$) CT26 tumors after treatment with anti-CTLA-4 and anti-PD-1 or isotype control antibodies as described for Figure 1G. (**C**) MLKL protein expression in Panc02.OVA pancreatic adenocarcinoma cells was assessed using western blotting. (**D-E**) C57BL6/J mice were inoculated with either WT or MLKL$^{-/-}$ Panc02.OVA pancreatic adenocarcinoma cells and were injected intraperitoneally with anti-CTLA-4, anti-PD-1 or isotype control antibodies. (**D**) Tumor growth of WT and MLKL$^{-/-}$ Panc02.OVA tumors in mice treated with anti-CTLA-4. (**E**) Overall survival of mice bearing either WT or MLKL$^{-/-}$ Panc02.OVA tumors after treatment with anti-PD-1. Data show mean tumor volume ± SEM or survival for n=5-10 mice per group that are either pooled from or representative of two independent experiments. Wild-type, WT.

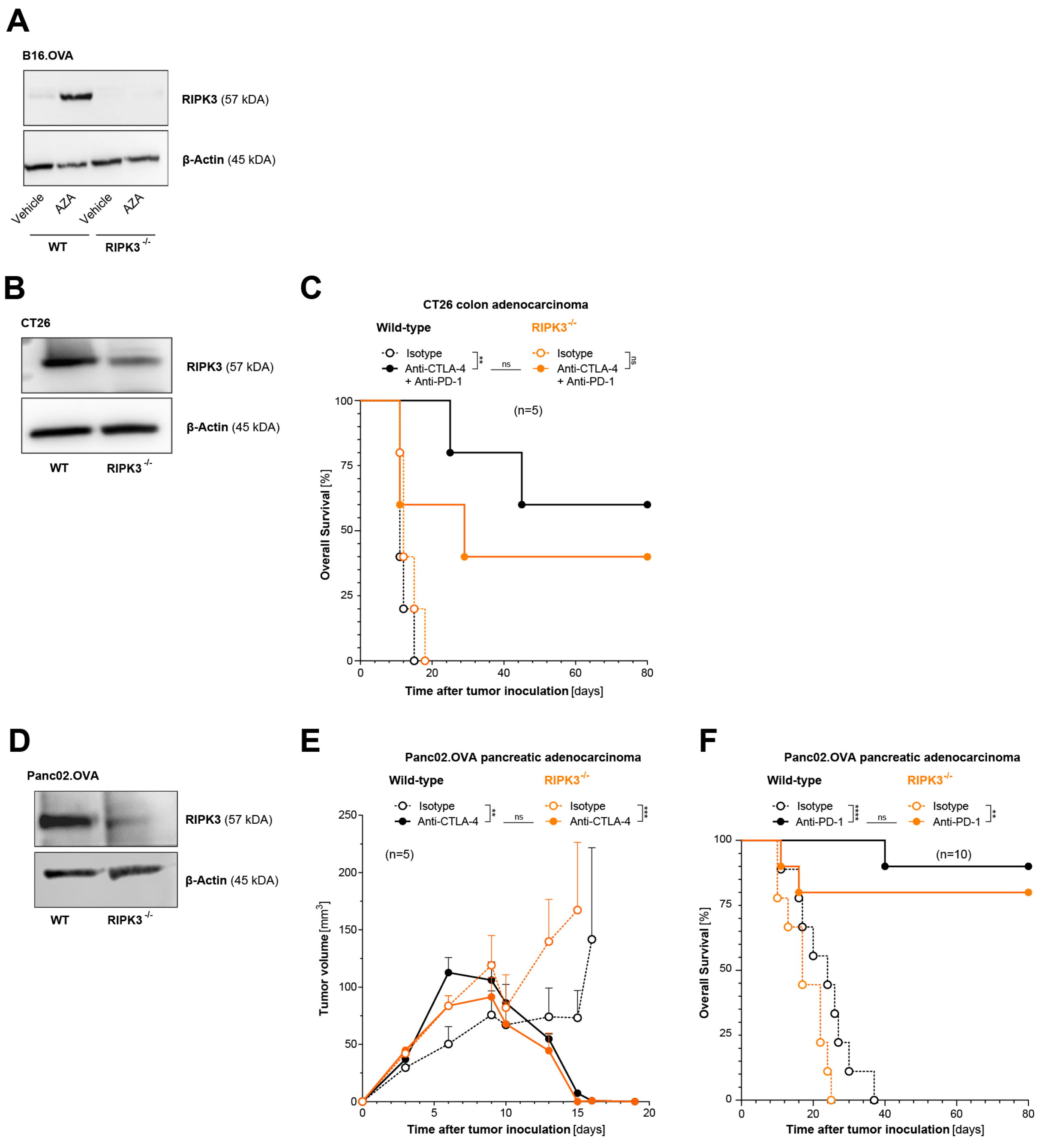

Figure S3: Tumor cell intrinsic loss of RIPK3 abrogates ICI immunotherapy in CT26 colon but not Panc02.OVA pancreatic adenocarcinoma. RIPK3 protein expression in gene engineered (**A**) B16.OVA and (**B**) CT26 cells was assessed using western blotting. Because of the artificial epigenetic downregulation of RIPK3 in B16.OVA cells under *in vitro* culture conditions, cells were pretreated with 4 µM of AZA for 72h to make differences in protein expression between wild-type and gene-engineered cells apparent. A sufficient level of genetic deletion of *Ripk3* in the CT26 cell line was confirmed by sequencing of extracted DNA (data not shown). (**C**) Overall survival of BALB/c mice bearing either wild-type or RIPK3$^{-/-}$ CT26 colon adenocarcinoma tumors after treatment with anti-CTLA-4 and anti-PD-1 as described for Figure 2D. (**D**) Western blot of RIPK3 protein expression in gene engineered Panc02.OVA

tumor cells. (**E-F**) C57BL6/J mice were inoculated with either WT or MLKL$^{-/-}$ Panc02.OVA pancreatic adenocarcinoma cells and were injected intraperitoneally with anti-CTLA-4, anti-PD-1 or isotype control antibodies. (**E**) Tumor growth of WT and RIPK3$^{-/-}$ Panc02.OVA tumors after treatment with anti-CTLA-4. (**F**) Overall survival of mice bearing either WT or RIPK3$^{-/-}$ Panc02.OVA tumor cells treated with anti-PD-1. Data show mean tumor volume ± SEM or survival for n=5-10 individual mice per group that are either pooled from or representative of two independent experiments.

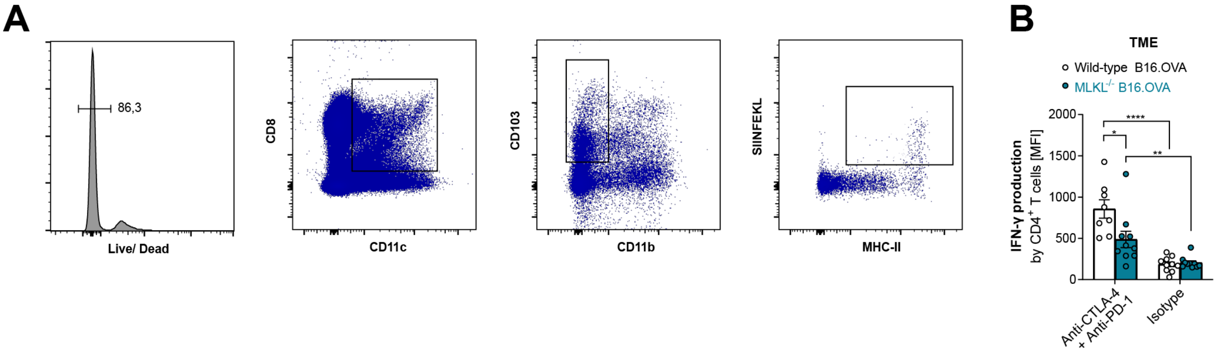

Figure S4: TME immunophenotyping - Defective necroptosis signaling in tumor cells impedes ICI-induced activation of tumor-infiltrating CD4$^+$ T cells. (**A**) Gating strategy to determine conventional dendritic cells type 1 (cDC1) in the tumor-draining lymph nodes (TdLN). Representative histograms are gated on cell death marker$^-$ CD8$^+$ CD11c$^+$ CD103$^+$ CD11b$^-$ MHC-II^{high} DCs. Within this population, specific cDC1 were defined as MHC-I SIINFEKL^{high} cells. (**B**) Expression of IFNγ in CD4$^+$ T cells in the TME presented as mean fluorescence intensity (MFI). Conventional dendritic cells type 1, cDC1. Mean fluorescence intensity, MFI. Tumor draining lymph nodes, TdLN.

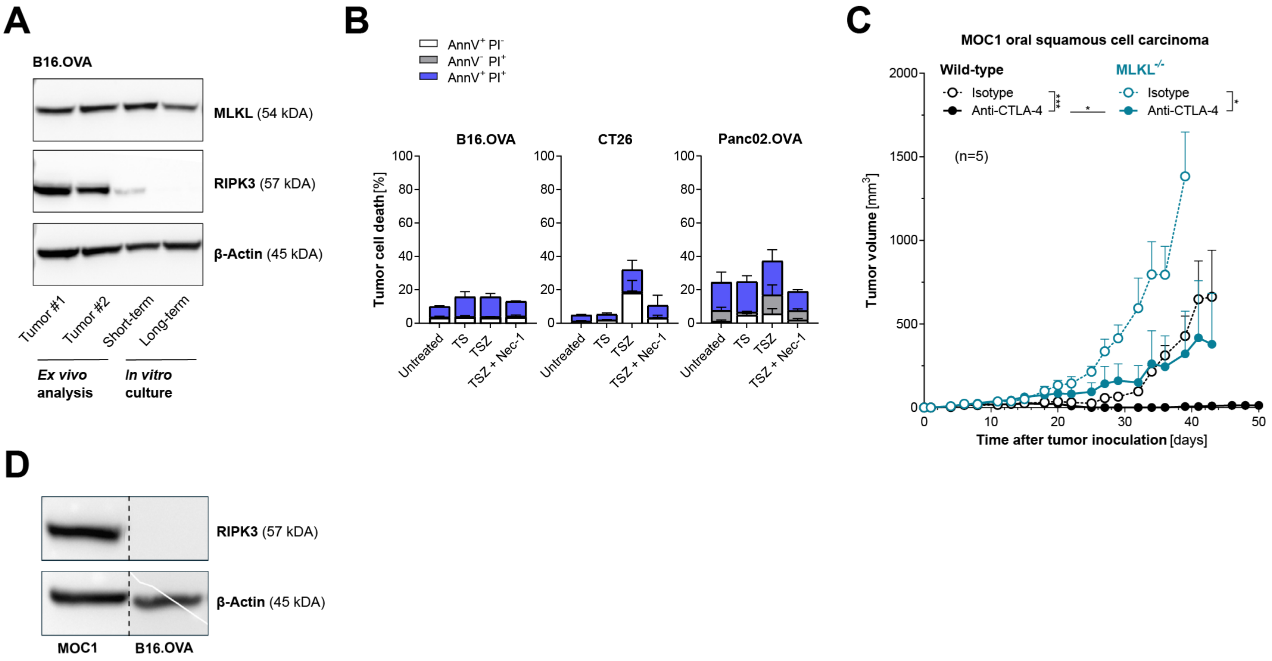

Figure S5: RIPK3 can be artificially downregulated in some tumor cell lines under *in vitro* culture conditions. (**A**) Expression of MLKL and RIPK3 as determined by western blot. *Ex vivo* analysis of two freshly isolated samples of B16.OVA tumors from C57BL/J mice (Tumor #1, Tumor #2), or analysis of B16.OVA cells after short-term or long-term *in vitro* cell culture. (**B**) Necroptosis was induced in B16.OVA, CT26 and Panc02.OVA cells as described for Figure 4, and cell death was assessed by annexin V and propidium iodide staining. (**C**) Tumor growth in C57BL6/J mice bearing either WT or MLKL$^{-/-}$ MOC1 oral squamous cell carcinoma tumors after treatment with anti-CTLA-4 and anti-PD-1. (**D**) RIPK3 protein expression in MOC1 and B16.OVA tumor cells under *in vitro* culture conditions was determined by western blot.

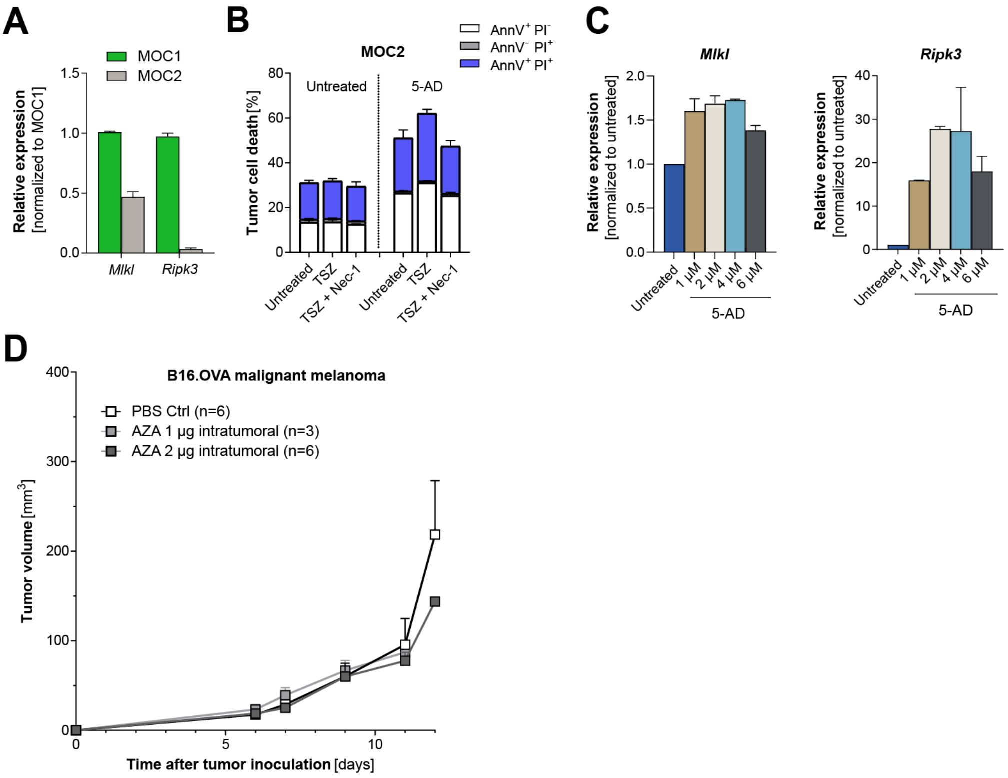

Figure S6: Exposure to 5-AD upregulated the transcriptional activity of *RIPK3* and *MLKL* and rendered the poorly immunogenic carcinoma cell line MOC2 susceptible to TSZ-induced necroptosis. (**A**) Relative gene expression of *Ripk3* and *Mlkl* in MOC1 and MOC2 tumor cell lines. Gene expression was determined by qPCR and normalized to expression in MOC1. (**B**) MOC2 cells were exposed to 1 µM aza-2′-deoxycytidine (5-AD) for 4 days. Necroptosis was induced in 5-AD-exposed and steady-state MOC2 cells as described for Figure 4. Induction of programmed cell death was assessed by Annexin V / propidium iodide staining and flow cytometry. (**C**) MOC2 cells were exposed for 4 days to different concentrations of 5-AD and gene expression of *Ripk3* and *Mlkl* was determined by qPCR. All *in vitro* date show mean ± SEM of triplicate samples that are representative of at least two independent experiments. (**D**) B16.OVA tumor growth in C57BL6/J mice treated with intratumoral injections of different doses of 5-azacytidine (AZA). The tumors were later extracted and used for qPCR analyses shown in Figure 5A. 5-AD, Aza-2′-deoxycytidine; AZA, 5-azacytidine.

A

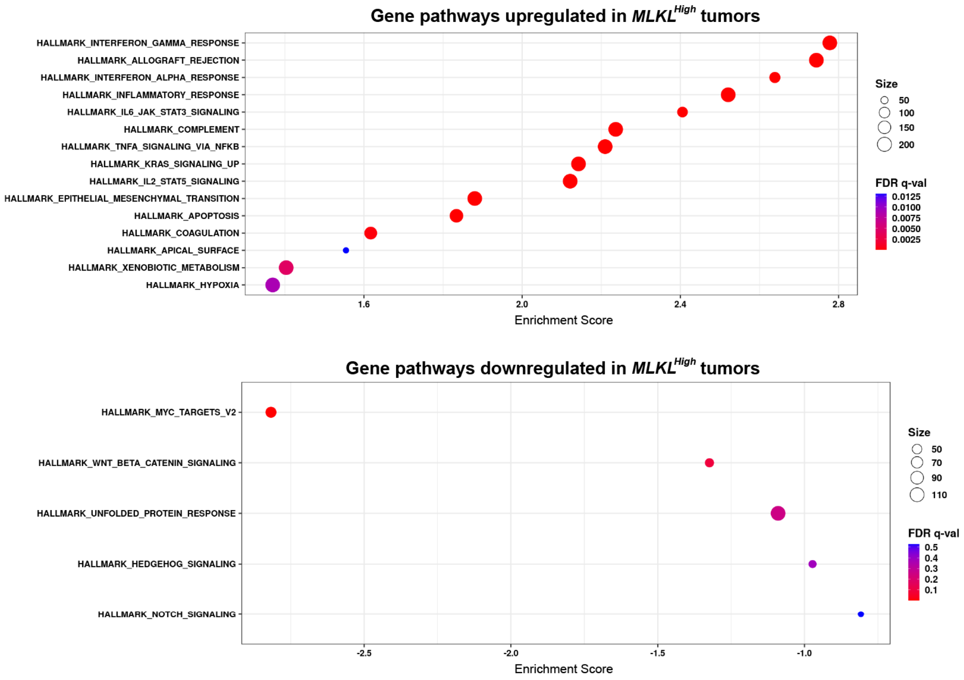

B

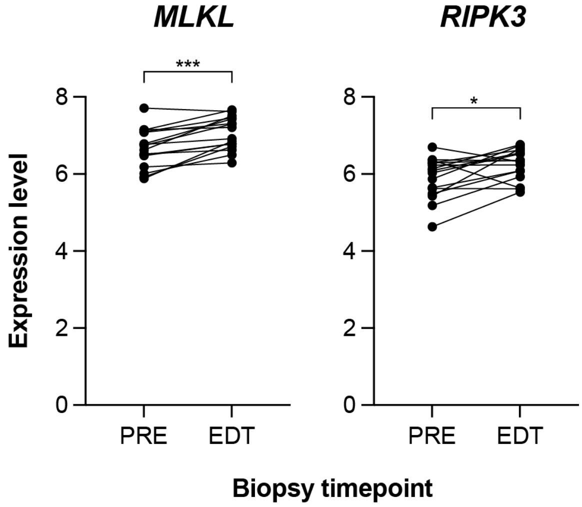

Figure S7: High transcriptional activity of *MLKL* in human melanoma tumors is associated with increased gene expression of inflammatory pathways. (A) Gene set enrichment analysis (GSEA) of differentially expressed genes (DEGs) in tumors with high versus low expression of *MLKL* in 472 melanoma patients from the TCGA databank. Fraction of DEGs in pathway is indicated by circle size, significance of enrichment by color. **(B)** Relative expression of indicated genes in paired pre-treatment (PRE) and on-treatment (EDT, early during treatment) tumor biopsies in patients with malignant melanoma undergoing ICI immunotherapy with anti-CTLA and/or anti-PD-1.

Variable	N	HR	95% CI	p-value
Sex				
male	264	1.000	(baseline)	
female	160	1.016	0.752 - 1.362	0.9158
Age, y				
<55	164	1.000	(baseline)	
55-65	107	1.190	0.817 - 1.716	0.3573
>65	153	1.671	1.182 - 2.364	0.0036
Tumor stage UICC				
I	93	1.000	(baseline)	
II	140	1.193	0.792 - 1.804	0.3994
III	169	1.925	1.331 - 2.815	0.0006
IV	22	3.504	1.643 - 6.791	0.0005
MLKL				
low	212	1.000	(baseline)	
high	212	0.511	0.379 - 0.686	<0.0001

Table S1: Low *MLKL* expression in melanoma biopsies is an independent risk factor for death. Multi-variable Cox regression analysis for overall survival in patients with malignant melanoma. HR, hazard ratio; CI, confidence interval; y, year.
